# Functional impacts of the ubiquitin–proteasome system on DNA damage recognition in global genome nucleotide excision repair

**DOI:** 10.1038/s41598-020-76898-2

**Published:** 2020-11-12

**Authors:** Wataru Sakai, Mayumi Yuasa-Sunagawa, Masayuki Kusakabe, Aiko Kishimoto, Takeshi Matsui, Yuki Kaneko, Jun-ichi Akagi, Nicolas Huyghe, Masae Ikura, Tsuyoshi Ikura, Fumio Hanaoka, Masayuki Yokoi, Kaoru Sugasawa

**Affiliations:** 1 Biosignal Research Center, Kobe University, Kobe, 657-8501 Japan; 2 Graduate School of Science, Kobe University, Kobe, 657-8501 Japan; 3 Division of Pathology, National Institute of Health Sciences, Kawasaki, 210-9501 Japan; 4 Institute of Experimental and Clinical Research, Catholic University of Louvain, 1200 Woluwe-Saint-Lambert, Brussels, Belgium; 5 Radiation Biology Center, Graduate School of Biostudies, Kyoto University, Kyoto, 606-8501 Japan; 6 National Institute of Genetics, Mishima, Shizuoka 411-8540 Japan

**Keywords:** Nucleotide excision repair, Ubiquitylation, Proteasome

## Abstract

The ubiquitin–proteasome system (UPS) plays crucial roles in regulation of various biological processes, including DNA repair. In mammalian global genome nucleotide excision repair (GG-NER), activation of the DDB2-associated ubiquitin ligase upon UV-induced DNA damage is necessary for efficient recognition of lesions. To date, however, the precise roles of UPS in GG-NER remain incompletely understood. Here, we show that the proteasome subunit PSMD14 and the UPS shuttle factor RAD23B can be recruited to sites with UV-induced photolesions even in the absence of XPC, suggesting that proteolysis occurs at DNA damage sites. Unexpectedly, sustained inhibition of proteasome activity results in aggregation of PSMD14 (presumably with other proteasome components) at the periphery of nucleoli, by which DDB2 is immobilized and sequestered from its lesion recognition functions. Although depletion of PSMD14 alleviates such DDB2 immobilization induced by proteasome inhibitors, recruitment of DDB2 to DNA damage sites is then severely compromised in the absence of PSMD14. Because all of these proteasome dysfunctions selectively impair removal of cyclobutane pyrimidine dimers, but not (6–4) photoproducts, our results indicate that the functional integrity of the proteasome is essential for the DDB2-mediated lesion recognition sub-pathway, but not for GG-NER initiated through direct lesion recognition by XPC.

## Introduction

Genomic DNA is continuously damaged by various endogenous and environmental factors. DNA repair alleviates diverse deleterious effects of such damage, including genomic instability and cell death. Nucleotide excision repair (NER) is a major DNA repair pathway with extremely broad substrate specificity^1,2^. The principal substrates for NER are (1) ultraviolet light (UV)-induced dipyrimidinic photolesions, such as cyclobutane pyrimidine dimer (CPD) and pyrimidine (6–4) pyrimidone photoproduct (6-4PP); (2) intrastrand crosslinks induced by cisplatin and other divalent compounds; and (3) bulky base adducts that can be caused by diverse chemical carcinogens^3^. In humans, hereditary defects in NER are implicated in several autosomal recessive disorders such as xeroderma pigmentosum (XP), which is clinically characterized by severe photosensitivity and a marked predisposition to skin cancer^4,5^.

A critical step in initiation of the NER reaction is lesion recognition, in which DNA sites with a relevant lesion must be distinguished from intact DNA, which is present in large excess. In mammalian global genome NER (GG-NER), the XP-related gene product XPC plays a key role in DNA lesion recognition^6^. The XPC protein forms a heterotrimeric complex in vivo with one of the two mammalian homologs of budding yeast RAD23 (RAD23A or RAD23B) and the Ca^2+^-binding EF-hand protein centrin-2^7,8^. This complex binds to the DNA duplex by recognizing the presence of disrupted or destabilized base pairs, rather than the specific chemical structure of any specific lesion^9–11^. These unique binding properties of XPC underlie the broad substrate specificity of GG-NER; however, the efficiency of repair initiated by XPC differs among lesion types depending on the extent of DNA helix distortion associated with each lesion. Typically, UV-induced 6-4PPs, which induce relatively large helix distortions, are efficiently recognized by XPC, whereas CPDs are poor substrates for XPC binding^12–14^. This problem is partially addressed by the presence of another lesion recognition factor dedicated to GG-NER, UV-damaged DNA–binding protein complex (UV-DDB), which consists of DDB1 and DDB2 (XPE) subunits^15,16^. Unlike XPC, UV-DDB specifically recognizes UV-induced photolesions (both 6-4PP and CPD) through direct interactions^17–20^, and then promotes recruitment of XPC to the damaged sites^21–23^. Because 6-4PPs can be processed through both UV-DDB–dependent and independent pathways, these lesions are repaired by GG-NER quite efficiently, even in the absence of UV-DDB. By contrast, removal of CPDs is much slower and more profoundly dependent on the UV-DDB–mediated lesion recognition pathway^24,25^.

The ubiquitin–proteasome system (UPS) plays crucial roles in regulation of myriad biological processes. The characteristic feature of this system is covalent conjugation of target proteins to one or more ubiquitin molecules, a process that relies on sequential actions of multiple enzymes (E1, E2, and E3)^26^ and leads to target degradation by the proteasome or functional alterations^27^. The lesion recognition process in GG-NER is intimately linked to UPS. For instance, RAD23, a component of the XPC complex, possesses a ubiquitin-like (UbL) domain and two ubiquitin-associated (UBA) domains^8,28,29^. In mammalian cells, RAD23 is much more abundant than XPC^30^ and, aside from its function in GG-NER, has been implicated in the regulation of degradation by the proteasome of ubiquitylated proteins^31–33^. Although both positive and negative roles for RAD23 have been proposed in protein degradation, interaction with RAD23 physically stabilizes XPC and protects it from degradation by the proteasome^34^. The two RAD23 orthologs are functionally indistinguishable in GG-NER^35^, but RAD23B is expressed at a much higher level than RAD23A; consequently, most XPC exists as a complex with RAD23B^36^.

In addition, UV-DDB associates in vivo with the Cullin-4 (CUL4)–RING ubiquitin E3 ligase^37,38^. This E3 complex, designated CRL4^DDB2^, is activated upon interaction of DDB2 with UV-induced DNA damage; the reported substrates of CRL4^DDB2^ include DDB2 itself, XPC, and histone proteins^39–41^. Our previous studies revealed that CRL4^DDB2^-mediated ubiquitylation can lead to degradation of DDB2, whereas ubiquitylation of XPC is mostly reversible^40^. Once UV-DDB associates with UV-induced DNA damage, timely recruitment of XPC suppresses the self-ubiquitylation and degradation of DDB2, thereby contributing to the sustainment of overall lesion recognition activity in the cell^42^. On the other hand, poly-ubiquitylated DDB2 is extracted from damaged sites by the ubiquitin-dependent segregase p97/VCP and targeted for degradation by the proteasome^43^. The sumoylation and RNF111-mediated ubiquitylation of XPC are also implicated in the UV-DDB–mediated lesion recognition pathway^44,45^. Thus, an intricate network of UPS and other related protein modifications ensures efficient recognition and removal of UV-induced DNA photolesions. However, it is not yet fully understood how the functions and dynamics of various UPS components influence each other during GG-NER.

In this study, we focused on the intracellular dynamics and localization of GG-NER–related UPS components, RAD23B, DDB2, and the proteasome. Our results revealed multifaceted impacts of proteasome dysfunctions on GG-NER of UV-induced DNA damage, beyond blockage of DDB2 degradation, providing novel insights into the roles of the UPS in regulation of DNA lesion recognition in GG-NER.

## Results

### RAD23B is recruited to DNA damage sites through multiple mechanisms

**Figure 1:**
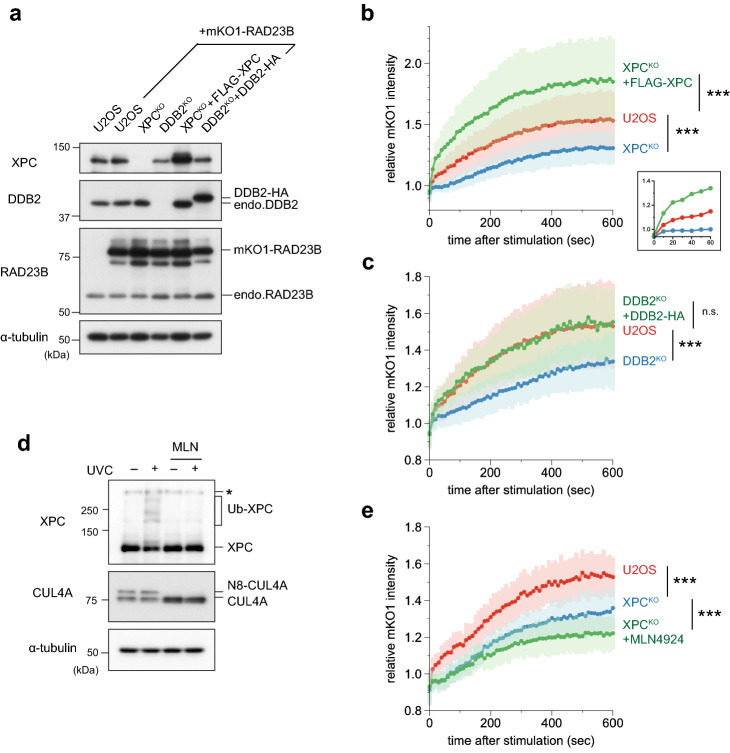
Real-time monitoring of recruitment of mKO1-RAD23B to local DNA damage. (**a**) Immunoblot analyses of endogenous and ectopically expressed XPC, DDB2, and RAD23B in the indicated cell lines. (**b,c)** Local DNA damage was induced in the indicated cell lines by three-photon absorption with an infrared femtosecond laser. Relative mKO1 intensities at damaged sites were quantitatively assessed over time. Inset in (**b**) shows enlargement of the graph in the early time range. The statistical significance assessed for the last time point is shown. *** *P* < 0.001. (**d**) Immunoblot analyses validating the effects of MLN4924. U2OS cells were pre-incubated for 2 h in the presence or absence of 1 µM MLN4924, and then irradiated with UVC at 10 J/m^2^ (or mock-irradiated) and further incubated for 1 h with or without MLN4924. Asterisk indicates non-specific reaction of the anti-XPC antibody. **e** The indicated cell lines were pre-treated for 2 h with or without 1 µM MLN4924, and recruitment of mKO1-RAD23B to local DNA damage was measured as in panels **b** and **c**. *** *P* < 0.001.

To investigate the roles of UPS-related factors in DNA damage recognition in GG-NER, we knocked out either endogenous *DDB2* or *XPC* in U2OS cells. These cells, as well as parental U2OS cells, were used to establish cell lines that stably expressed RAD23B fused to the C-terminus of monomeric Kusabira Orange 1 (mKO1), a fluorescent protein (designated as mKO1-RAD23B). Expression of XPC, DDB2, and RAD23B proteins in these cell lines was validated by immunoblot analysis (Fig. 1a). In all cell lines, mKO1-RAD23B was overexpressed relative to the endogenous protein. A confocal laser scanning microscope was customized to monitor protein dynamics in response to local DNA damage generated by stimulation comparable to 260-nm UVC irradiation^46^. Using this system, fluorescently-labeled DDB2 and XPC rapidly accumulated in irradiated areas, confirming generation of DNA photolesions such as CPDs and 6-4PPs (Supplementary Fig. 1a and b).

Upon induction of local DNA damage in the wild-type U2OS background, mKO1-RAD23B accumulated at damaged sites (Supplementary Fig. 1c). In XPC^KO^ cells, this accumulation of mKO1-RAD23B was significantly reduced (Fig. 1b), as expected from the notion that XPC associates with RAD23B in vivo and is recruited to DNA damage as a complex. Complementation with exogenous XPC expression robustly stimulated the accumulation of mKO1-RAD23B to even higher levels than those observed in wild-type U2OS cells (Fig. 1b). This is likely because exogenous XPC was overexpressed relative to the endogenous protein (Fig. 1a). Because the RAD23B protein is expressed much more abundantly than XPC in a cell, most of the overexpressed XPC likely bound to RAD23B to form functional lesion recognition complexes. Therefore, it is conceivable that the enhanced recruitment of RAD23B, at least partly, was a consequence of the increased number of XPC molecules that were recruited to damaged sites. Similarly, in DDB2^KO^ cells, the accumulation of mKO1-RAD23B was also compromised and restored to control levels by complementation with exogenously expressed DDB2 (Fig. 1a,c). These results are consistent with the model that DDB2 facilitates recruitment of XPC, together with RAD23B, to UV-induced DNA damage.

Overall, the results described above recapitulate the established roles of XPC-RAD23B and UV-DDB in recognition of UV-induced photolesions in GG-NER. Notably, however, mKO1-RAD23B accumulated at sites of DNA damage at a non-negligible level, even in the absence of XPC (Fig. 1b). This XPC-independent recruitment of mKO1-RAD23B was also observed following conventional local UVC irradiation through isopore membrane filters (Supplementary Fig. 2). Intriguingly, XPC-independent mKO1-RAD23B accumulation occurred with a significant time lag relative to stimulation (Fig. 1b, inset), suggesting that it depends on events that take place in or near the sites of DNA damage. The most likely candidate for a mediator of such events was CRL4^DDB2^-mediated ubiquitylation. Hence, we treated XPC^KO^ cells with MLN4924, a pan-inhibitor of Cullin-RING ubiquitin E3 ligases (CRLs)^47^, and analyzed the effects on recruitment of mKO1-RAD23B in response to DNA damage. Immunoblot analyses confirmed that MLN4924 treatment suppressed not only conjugation of NEDD8 to CUL4A, but also UV-induced ubiquitylation of XPC, as expected (Fig. 1d). Under such conditions, the XPC-independent accumulation of mKO1-RAD23B was also attenuated (Fig. 1e). Taken together, these results indicate that RAD23B can be recruited to sites of UV-induced DNA damage through at least two pathways: recruitment as a subunit of the XPC damage recognition complex, and ubiquitylation induced by CRL4^DDB2^ independently of XPC.

### Proteasome inhibitors alter the dynamics and localization of DDB2

**Figure 2:**
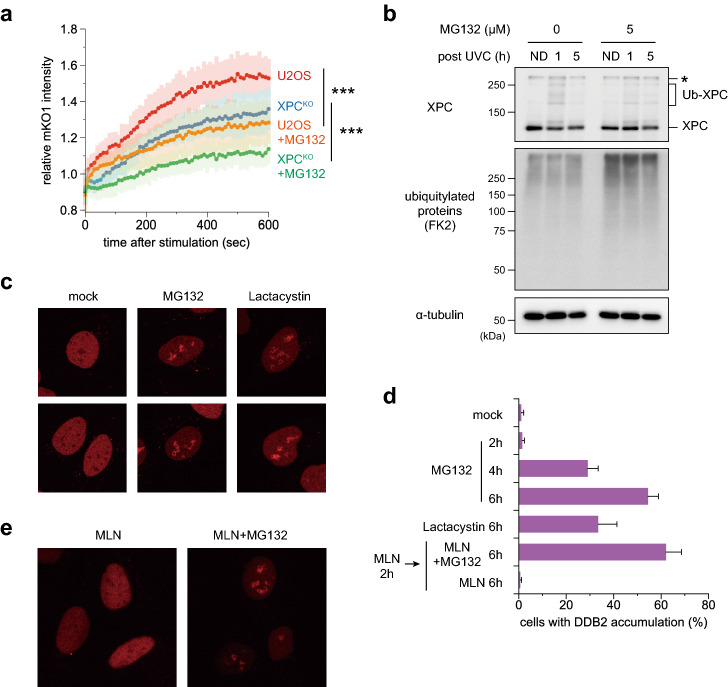
Inhibition of the proteasome affects dynamics and localization of GG-NER factors. (**a**) U2OS and XPC^KO^ cells were pre-treated for 2 h with 5 µM MG132 or DMSO (solvent only). Recruitment of mKO1-RAD23B to local DNA damage was assessed as in Fig. 1. *** *P* < 0.001. (**b**) Immunoblot analyses of the effects of MG132. U2OS cells were pre-cultured for 2 h in the presence or absence of 5 µM MG132, irradiated with UVC at 10 J/m^2^, and further incubated for the indicated times with or without MG132. Asterisk indicates non-specific reaction of the anti-XPC antibody. ND: unirradiated control cells. (**c**) Representative images of the DDB2-mKO1 localization in the cells, which were mock-treated or treated for 6 h with 5 µM MG132 or 10 µM lactacystin. (**d**) Percentages of cells showing subnuclear DDB2-mKO1 accumulation after the indicated treatments. At least three independent experiments were carried out, and more than 300 cells in total were examined for each treatment. **e** Cells expressing DDB2-mKO1 were pre-incubated for 2 h in the presence of 1 µM MLN4924, and subsequently treated for 6 h with MLN4924 only (left) or with MLN4924 plus MG132 (right).

Considering the proposed functional links between RAD23 and UPS, we next examined the impacts of proteasome inhibition. In the presence of the proteasome inhibitor MG132, recruitment of mKO1-RAD23B to DNA damage sites was significantly reduced (Fig. 2a). This effect was observed regardless of the presence or absence of XPC, suggesting that the DDB2-mediated lesion recognition pathway is affected more profoundly by MG132 than direct recognition by XPC. MG132 treatment resulted in accumulation of ubiquitylated proteins, as expected, whereas ubiquitylation of XPC induced transiently by UVC irradiation was weakened (Fig. 2b). This is not surprising; we previously reported that a major fraction of ubiquitylated XPC is not subjected to degradation by the proteasome^40^. Reduced XPC ubiquitylation could be explained, at least partly, by depletion of free ubiquitin^48,49^, while such a defect in CRL4^DDB2^-mediated ubiquitylation may cause reduced RAD23B recruitment (Fig. 2a), as observed with MLN4924 (Fig. 1e).

To understand the effects of RAD23 and proteasome inhibition in more detail, we examined the intracellular dynamics and localization of DDB2. For this purpose, DDB2 fused to the N-terminus of mKO1 (DDB2-mKO1) was stably expressed in U2OS cells. Upon local UVC stimulation, DDB2-mKO1 rapidly accumulated to damaged sites, which peaked around 1 min of post-stimulation and then decreased gradually (Supplementary Fig. 1a and 3a). In the RAD23B^KO^ background, the DDB2-mKO1 accumulation was significantly enhanced, suggesting that RAD23B suppresses the recruitment of DDB2 or promotes removal of DDB2 from DNA damage sites. By contrast, 2-h pre-treatment with MG132 marginally reduced the DDB2-mKO1 recruitment in U2OS cells, although the difference was not statistically significant (Supplementary Fig. 3b). Fluorescence recovery after photobleaching (FRAP) analyses revealed a significant delay of fluorescence recovery, indicating that DDB2 mobility was reduced by inhibition of the proteasome (Supplementary Fig. 3c). Furthermore, prolonged proteasome inhibition induced remarkable re-localization of DDB2 within the nucleus. Under normal culture conditions, DDB2-mKO1 localized throughout the nucleus, except in certain subnuclear domains (Fig. 2c, left panels). Notably, after 6-h treatment with MG132 or lactacystin, another proteasome inhibitor, DDB2-mKO1 accumulated densely in the nucleus, often with fibrous morphology (Fig. 2c, middle and right panels). This characteristic DDB2 localization was barely observed after 2-h incubation with MG132, and then gradually increased over time (Fig. 2d). It should be noted that, like the reduction in mobility observed in Supplementary Fig. 3c, this DDB2 accumulation occurred without DNA damage from exogenous sources, such as UV. In addition, co-treatment with MLN4924 did not prevent accumulation (Fig. 2d,e), indicating that self-ubiquitylation of DDB2 mediated by CRL4^DDB2^ is not necessary for this phenomenon. FRAP analyses showed that the accumulated DDB2-mKO1 molecules were virtually immobile (Supplementary Fig. 4a and b). However, after withdrawal of the inhibitor, the accumulated DDB2-mKO1 gradually dissipated (Supplementary Fig. 4c), indicating that the structure formed by DDB2-mKO1 was distinct from an insoluble aggregate.

**Figure 3:**
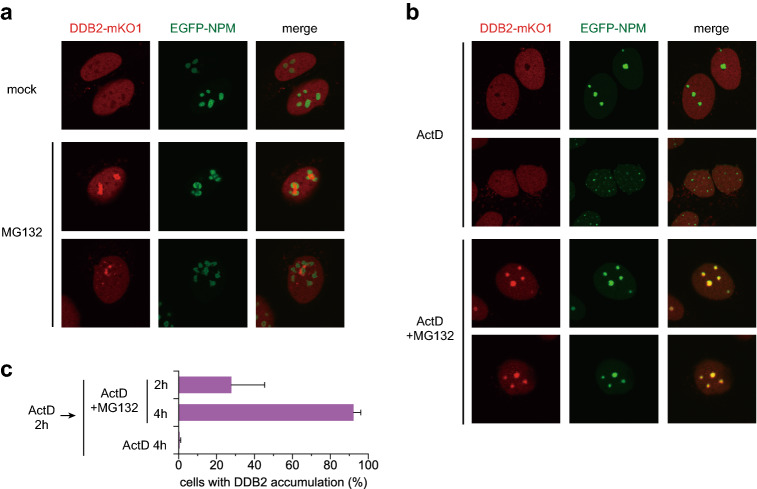
MG132-induced relocalization of DDB2 is related to nucleoli. (**a**) Cells expressing both DDB2-mKO1 and EGFP-NPM were mock-treated or incubated for 6 h in the presence of 5 µM MG132. Live-cell images were acquired with a confocal laser scanning microscope. (**b)** After 2-h pre-incubation with 40 nM ActD, cells were further treated for 4 h with ActD only or with ActD plus MG132. (**c)** Percentages of the cells showing DDB2-mKO1 accumulation were quantified as in Fig. 2d.

### Subnuclear localization of DDB2 is related to nucleolar functions

Inhibition of the proteasome results in accumulation of misfolded proteins, which form aggregates in the cytoplasm as well as the nucleus^50,51^. Notably, several nuclear proteasome target proteins form aggregates in nucleoli^52^. Based on these notions, we next examined the possible relationship between DDB2 re-localization and nucleoli. To this end, we stably co-expressed an N-terminal EGFP fusion of the nucleolar marker protein nucleophosmin (EGFP-NPM) along with DDB2-mKO1 in U2OS cells. Under normal culture conditions, DDB2-mKO1 and EGFP-NPM localized within the nucleus in a mutually exclusive fashion (Fig. 3a). After 6-h treatment with MG132, the nucleoli underwent substantial morphological changes, as described previously^52^, whereas the accumulated DDB2-mKO1 and EGFP-NPM appeared side-by-side, but still did not overlap (Fig. 3a). Often, the DDB2 and NPM signals appeared to surround one another.

These findings prompted us to examine whether integrity of nucleolar functions affects the localization of DDB2. Treatment with actinomycin D (ActD) inhibits transcription by RNA polymerase I (RNAPI), thereby inducing segregation of nucleolar components and formation of structures called nucleolar caps^53^. When cells co-expressing DDB2-mKO1 and EGFP-NPM were treated with ActD alone, disintegration of NPM signals was observed in some cells, but accumulation of DDB2 did not occur, and the mutually exclusive localization of DDB2 and NPM was maintained (Fig. 3b). However, simultaneous treatment with ActD and MG132 dramatically facilitated DDB2 accumulation, resulting in co-localization with NPM (Fig. 3b,c). These results suggest that the functions of the proteasome and nucleoli cooperatively regulate the subnuclear localization of DDB2.

**Figure 4:**
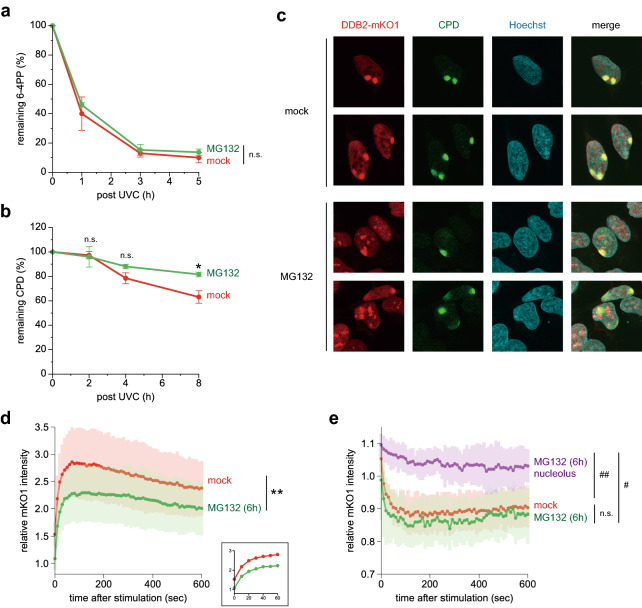
Inhibition of proteasome activity compromises GG-NER. (**a**,**b**) U2OS cells were pre-incubated for 2 h in the presence or absence of 5 µM MG132. The cells were then irradiated with UVC at 10 J/m^2^ (**a**) or 2 J/m^2^ (**b**), and further incubated for the indicated times with or without MG132. Amounts of 6-4PPs (**a**) and CPDs (**b**) remaining in genomic DNA were quantified by enzyme-linked immunosorbent assay with lesion-specific antibodies. * *P* < 0.05. (**c**) Cells expressing DDB2-mKO1 were pre-incubated for 6 h in the presence or absence of 5 µM MG132, and then irradiated with UVC at 100 J/m^2^ through isopore membrane filters. After a 10-min incubation with or without MG132, the cells were fixed and co-stained with anti-mKO2 and anti-CPD antibodies, and visualized with Alexa Fluor 594- and 488-conjugated secondary antibodies, respectively. Nuclear DNA was counter-stained with Hoechst 33342. (**d**) Cells expressing DDB2-mKO1 were pre-treated for 6 h with 5 µM MG132. The cells with nucleolar accumulation of DDB2 were chosen, and local UVC stimulation was applied to the areas outside the nucleoli. Recruitment of DDB2-mKO1 to the damaged sites was quantitatively assessed and compared with the data obtained with mock-treated cells. Inset shows enlargement of the graph in the early time range. The statistical significance assessed for the last time point is shown. ** *P* < 0.01. (**e**) Cells were treated as in (**d**), and fluorescence intensities of DDB2-mKO1 in the unstimulated areas were monitored. For MG132-treated cells, the areas with (purple line) or without (green line) the DDB2 accumulation were examined. # *P* < 1 × 10^–8^, ## *P* < 1 × 10^–10^.

We next addressed how proteasome inhibition affects the process of GG-NER. After U2OS cells were irradiated with UVC, removal of photolesions from the entire genome was quantitatively assessed in the presence or absence of MG132. Among UV-induced photolesions, 6-4PPs are repaired quite rapidly. Indeed, approximately 90% of 6-4PPs were removed within 3 h, and this rate was not significantly affected by treatment with MG132 (Fig. 4a). By contrast, repair of CPDs is relatively slow and was further delayed in the presence of MG132, especially at later time points (Fig. 4b). Furthermore, local UVC irradiation through isopore membrane filters revealed that after 6-h treatment with MG132, a fraction of DDB2-mKO1 was still recruited to damage sites, whereas previously formed accumulations of DDB2-mKO1 persisted (Fig. 4c). Next we quantitatively assessed kinetics of the DDB2-mKO1 recruitment to DNA damage sites upon local UVC stimulation. In line with the results of immunofluorescence staining, rapid recruitment of DDB2-mKO1 was observed regardless with or without MG132 treatment (Fig. 4d). However, the cumulative level of DDB2-mKO1 recruitment was significantly reduced by the 6-h treatment with MG132 in comparison with mock-treated, control cells. In this experiment, fluorescence intensities in non-damaged areas were also monitored, which revealed that the level of DDB2-mKO1 was reciprocally reduced upon induction of local DNA damage in the control cells (Fig. 4e, red line). Non-nucleolar areas in MG132-treated cells showed similar kinetics of the decrease in fluorescence intensity (Fig. 4e, green line), while accumulation of DDB2-mKO1 at the periphery of nucleoli remained much more stably (purple line). Taken together, our results indicate that prolonged inhibition of proteasome activity leads to re-localization of DDB2, which restricts the availability of DDB2 for initiation of GG-NER and affects the relatively slow repair of CPDs more profoundly than that of 6-4PPs.

### Depletion of a proteasome subunit has distinct effects on GG-NER

**Figure 5:**
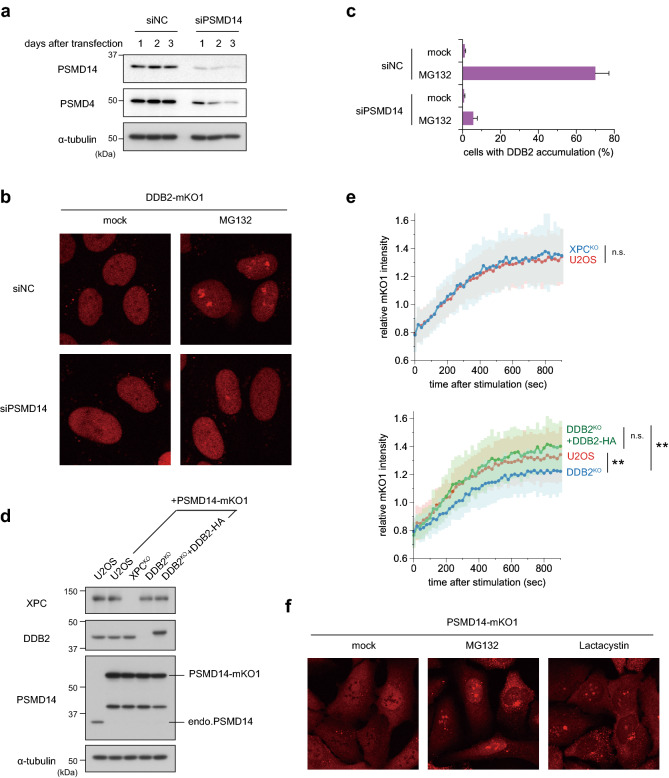
MG132-induced DDB2 accumulation depends on the proteasome subunit PSMD14. (**a**) Immunoblot analyses validating depletion of the proteasome subunits. U2OS cells were transfected with negative control siRNA (siNC) or siRNA targeting PSMD14 (siPSMD14), and cell lysates were prepared after incubation for the indicated number of days. All subsequent experiments were performed on the second day of post-transfection. (**b,c)** Cells expressing DDB2-mKO1 were treated with siNC or siPSMD14, and then incubated for 6 h in the presence or absence of 5 µM MG132. Based on live-cell images acquired with a confocal laser scanning microscope (**b**), percentages of cells with subnuclear DDB2 accumulation were measured as in Fig. 2d. **(d)** Immunoblot analyses validating expression of the XPC, DDB2, and PSMD14 proteins in the indicated cell lines. **(e)** Recruitment of PSMD14-mKO1 to local DNA damage was assessed quantitatively in the indicated cell lines. ** *P* < 0.01. (**f)** Representative live-cell images of PSMD14-mKO1 localization in cells that were mock-treated or treated for 6 h in the presence of 5 µM MG132 or 10 µM lactacystin.

Given that inactivated proteasomes are present in cells during treatment with proteasome inhibitors, we next depleted a proteasome subunit and examined the effects of this intervention on GG-NER. To this end, we chose PSMD14, a component of the 19S regulatory particle, which possesses deubiquitylation activity and participates in salvaging ubiquitin molecules upon degradation of target proteins^54^. When we treated U2OS cells with siRNA targeting PSMD14, we noticed that levels of not only PSMD14 itself, but also PSMD4, were reduced, indicating that the entire 19S regulatory particle was destabilized (Fig. 5a). As predicted, reduced PSMD14 expression impaired cell proliferation^55^; consequently, a substantial number of enlarged cells were observed after a 2-day incubation with siRNA. However, this treatment did not lead to significantly reduced mobility of DDB2-mKO1 in FRAP analysis (Supplementary Fig. 3d) or re-localization of DDB2-mKO1 within the nucleus (Fig. 5b). Notably, the MG132-induced accumulation of DDB2-mKO1 was strongly suppressed by treatment with siRNA targeting PSMD14 (Fig. 5b,c), which suggests the involvement of inactive proteasomes in DDB2 re-localization.

These results prompted us to examine the intracellular dynamics and localization of the proteasome subunit. PSMD14 fused to the N-terminus of mKO1 (PSMD14-mKO1) was stably expressed in U2OS cells with wild-type, XPC^KO^, or DDB2^KO^ background (Fig. 5d). In these cell lines, PSMD14-mKO1 was overexpressed, whereas expression of endogenous PSMD14 was almost completely suppressed. Because these cells proliferated normally, it is likely that ectopically expressed PSMD14-mKO1 displaced the endogenous protein to form functional 19S regulatory particles. In these cell lines, we observed recruitment of PSMD14-mKO1 to DNA damage sites, albeit with slower kinetics of accumulation than DDB2, XPC, and RAD23B (Fig. 5e and Supplementary Fig. 1d). Immunofluorescence staining after local UVC irradiation through isopore membrane filters also revealed that PSMD14 colocalized with DDB2 in the damaged areas (Supplementary Fig. 5). Notably, this recruitment of PSMD14-mKO1 was dependent on the presence of functional DDB2, but not XPC (Fig. 5e). Under normal culture conditions, PSMD14-mKO1 localized in both the cytoplasm and nucleus (Fig. 5f and Supplementary Fig. 6a). As with DDB2, PSMD14 appeared to be excluded from the nucleoli, whereas some bright, dot-like localization was also discerned in the cytoplasm. After 6-h treatment with MG132 or lactacystin, however, we observed accumulation of PSMD14 in the vicinity of the nucleoli, as well as an increase in the abundance of cytoplasmic dots. Moreover, PSMD14 co-localized with nucleolar signals in the presence of both ActD and MG132 (Supplementary Fig. 6a), which was also highly reminiscent of the behaviors of DDB2 (Fig. 3). mKO1-RAD23B did not show similar accumulation at the periphery of nucleoli (Supplementary Fig. 6b), excluding the possibility that the observed re-localization of DDB2 and PSMD14 was due to an mKO1-related artifact. Taken together, our results suggest that inhibition of proteasome activity leads to accumulation of proteasome components within the nucleus, which sequesters DDB2 and prevents it from participating in GG-NER.

### Roles of PSMD14 activity in GG-NER

**Figure 6:**
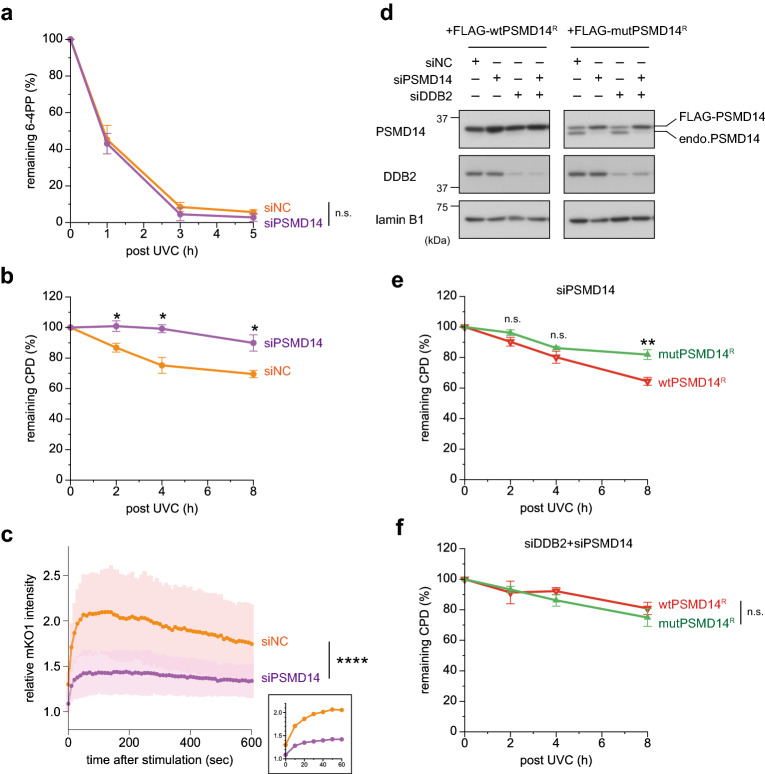
Presence of a functional proteasome is required for efficient CPD repair. (**a**,**b**) U2OS cells treated with siNC or siPSMD14 were used to assess repair kinetics of 6-4PPs (**a**) and CPDs (**b**) as in Fig. 4. * *P* < 0.05. (**c)** Cells expressing DDB2-mKO1 were treated with siNC or siPSMD14, and recruitment of DDB2-mKO1 to local DNA damage was quantitatively assessed. The statistical significance assessed for the last time point is shown. **** *P* < 1 × 10^–4^. (**d)** Immunoblot analyses validating the effects of siRNAs and the resistance of the ectopically-expressed PSMD14^R^ proteins to the siPSMD14. (**e,f)** Cells expressing wtPSMD14^R^ or mutPSMD14^R^ were treated with siPSMD14 only (**e**) or siDDB2 plus siPSMD14 (**f**). After UVC irradiation at 2 J/m^2^, repair kinetics of CPDs were assessed. ** *P* < 0.01.

Finally, we examined the effects of perturbed PSMD14 functions on GG-NER. When PSMD14 was depleted of U2OS cells with siRNA, repair of CPDs, but not 6-4PPs, was significantly impaired (Fig. 6a,b). Unexpectedly, upon local UVC stimulation, depletion of PSMD14 severely reduced recruitment of DDB2-mKO1 to DNA damage sites (Fig. 6c), which can explain for the selective repair defect of CPDs. Although inhibition of proteasome activity and depletion of PSMD14 had distinct impacts on dynamics and localization of DDB2, both resulted in similar outcomes in GG-NER. To exclude possible off-target effects of the siRNA, we carried out rescue experiments. For this purpose, an siRNA-resistant allele of wild-type PSMD14 (wtPSMD14^R^) was fused to the FLAG-tag and stably expressed in U2OS cells. Another transformed cell line, established for comparison, stably expressed siRNA-resistant mutant PSMD14 (mutPSMD14^R^), which lacked deubiquitylation activity due to amino acid substitutions in the JAMM (JAB1/MPN/Mov34 metalloenzyme) motif^56^. As observed with the mKO1-fusion protein (Fig. 5d), ectopic expression of wtPSMD14^R^ resulted in expulsion of most endogenous PSMD14 from the 19S regulatory particles (Fig. 6d, left panel). As expected, expression of wtPSMD14^R^ protein was resistant to siRNA and even increased slightly, presumably due to compensation for loss of residual endogenous PSMD14. On the other hand, mutPSMD14^R^ was expressed at a level comparable to endogenous PSMD14, and nearly complete displacement was achieved by treatment with siRNA (Fig. 6d, right panel). After this siRNA treatment, cells expressing mutPSMD14^R^ exhibited a significant delay in repair of CPDs relative to control cells expressing wtPSMD14^R^ (Fig. 6e). Notably, this difference was substantially attenuated when expression of endogenous DDB2 was simultaneously suppressed (Fig. 6f). Taken together, these results indicate that intact functions of PSMD14 (presumably as the 19S regulatory particle) are crucial, particularly for the UV-DDB–mediated DNA lesion recognition pathway of GG-NER. By contrast, the PSMD14 dysfunction has a much weaker impact on direct lesion recognition by XPC, which is still able to initiate efficient removal of 6-4PPs, but not CPDs.

## Discussion

**Figure 7:**
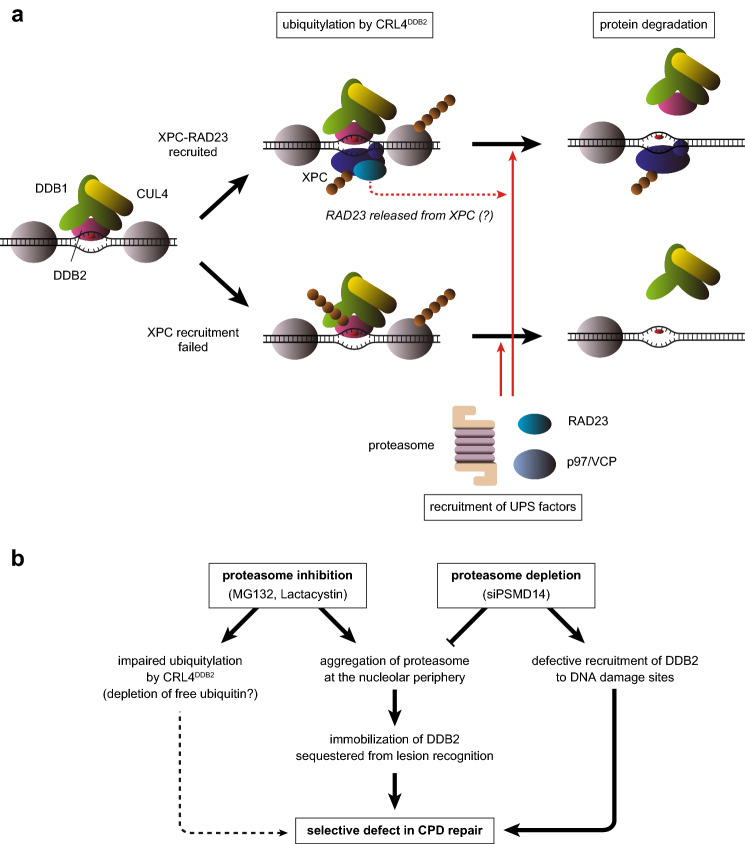
Schematic representation of functional impacts of UPS on GG-NER. (**a**) Model for roles of UPS factors in the GG-NER process. The CRL4^DDB2^ E3 ligase bound to a DNA lesion ubiquitylates DDB2, XPC, and other proteins nearby (such as histones). Successful recruitment of XPC-RAD23 competitively suppresses ubiquitylation and degradation of DDB2. UPS factors such as the proteasome, RAD23, and p97/VCP are then recruited to degrade poly-ubiquitylated substrates. (**b)** Impacts of proteasome dysfunctions revealed by this study.

Using real-time monitoring of fluorescently-labeled proteins following local UVC stimulation, we showed that RAD23 can be recruited to sites of DNA damage, not only as part of the XPC lesion recognition complex but also by an XPC-independent mechanism. The latter phenomenon was suppressed by the CRL inhibitor MLN4924, suggesting an essential role for CRL4^DDB2^-mediated ubiquitylation of particular proteins. RAD23 possesses a UbL domain at the N-terminus that interacts with the 19S regulatory particle of the proteasome via PSMD4^57^, whereas two UBA domains in a more C-terminal region of the protein interact with ubiquitin^58,59^. Based on this characteristic structure–function relationship, RAD23 is considered to be a shuttle factor that recruits ubiquitylated substrate proteins to the 26S proteasome^31,32^. Indeed, when FLAG-tagged PSMD14^R^ expressed in U2OS cells was immunoprecipitated, we detected co-precipitation of ubiquitylated proteins as well as RAD23B (Supplementary Fig. 7). Not only the interaction with ubiquitylated proteins, but also that with RAD23B, were more pronounced with mutPSMD14^R^ than with wtPSMD14^R^, which appears consistent with the role for RAD23 as a proteasome shuttle factor. Therefore, it is likely that degradation of one or more ubiquitylated proteins, presumably including DDB2, is accompanied by XPC-independent RAD23 recruitment. This notion is consistent with our data showing that the proteasome subunit PSMD14 is also recruited to damaged sites in a DDB2-dependent manner (Fig. 5e). Notably, our previous study using a patient-derived XPC-deficient cell line showed that XPC stochastically suppresses UV-induced self-ubiquitylation and degradation of DDB2^42^. In line with this notion, the UV-induced destabilization of DDB2 was more pronounced in the XPC^KO^ cells used in the present study than in parental U2OS cells (Supplementary Fig. 8). Moreover, depletion of RAD23B is known to destabilize XPC^34,36^. Our RAD23B^KO^ cells indeed exhibited not only reduced XPC expression, but also an intermediate level of DDB2 stability between U2OS and XPC^KO^ cells (Supplementary Fig. 8). Although we believe that DDB2 degradation and XPC-independent RAD23 recruitment also occur in normal cells, we might observe enhancement of these processes in the XPC^KO^ background of the cell line. On the other hand, it was reported that at least part of XPC bound to damaged chromatin exists as a RAD23-free form^60,61^. In addition to RAD23 recruited by itself, RAD23 released from XPC could play a role in regulation of protein degradation at the damage sites (Fig. 7a).

Given that RAD23 is directly involved in degradation of ubiquitylated DDB2, it remains unclear when and where DDB2 is degraded by the proteasome. Biochemical studies revealed that poly-ubiquitylation of DDB2 abrogates its ability to bind damaged DNA^40^, suggesting that it can dissociate spontaneously from the bound lesion upon ubiquitylation. On the other hand, the p97/VCP segregase is implicated in efficient removal of ubiquitylated DDB2 from damaged sites and its subsequent degradation by the proteasome^43^. RAD23 and other proteasome shuttle factors function downstream of p97/VCP^62^, which then deliver the ubiquitylated DDB2 to the 26S proteasome. However, these findings do not necessarily mean that ubiquitylated DDB2 is completely released from the lesion site prior to degradation, and we cannot exclude the possibility that DDB2 is degraded on site immediately after self-ubiquitylation. In this regard, it is notable that accumulation of DDB2 to local DNA damage was significantly enhanced in the absence of RAD23B (Supplementary Fig. 3a). This could be explained by accumulation of ubiquitylated DDB2, which was prevented from delivery to the proteasome. Alternatively, it is also possible that recruitment of XPC facilitates displacement of DDB2 from damage sites. Because the XPC expression level was reduced by depletion of RAD23B (Supplementary Fig. 8a), more DDB2 waiting for recruitment of XPC could be retained at damaged sites for certain time periods. We think that both mechanisms may contribute to the enhanced DDB2 accumulation in a concerted manner.

Our results show that PSMD14-mKO1 accumulates in response to local UVC stimulation, but with slower kinetics than DDB2, XPC, and RAD23B. Notably in this regard, the proteasome is one of the most abundant protein factors in a cell, so even weak accumulation may indicate the presence of substantial numbers of molecules at damaged sites. This gradual increase in fluorescence resembles the XPC-independent accumulation of RAD23B, suggesting that recruitment of the proteasome depends on certain reactions occurring at lesion sites, most likely ubiquitylation. Furthermore, our results revealed that a substantial fraction of PSMD14 accumulation is independent of DDB2. By blocking DNA replication and transcription, UV-induced DNA damage can provoke various responses in addition to GG-NER; for instance, transcription-coupled NER (TC-NER), translesion DNA synthesis, and double-strand break repair, all of which are associated with UPS-mediated protein degradation. In addition, it also should be noted that three-photon absorption following infrared laser irradiation has the potential to induce types of DNA damage other than canonical NER substrates^63^. Nevertheless, the DDB2-dependent recruitment of PSMD14 strongly supports the involvement of the proteasome in the process of GG-NER.

In this study, we showed that treatment with proteasome inhibitors affects the mobility and localization of DDB2 in the nucleus. Under normal culture conditions, DDB2 appeared to localize throughout the nucleus except in nucleoli. After relatively short treatment (2 h), FRAP analyses revealed a significant reduction in the mobility of DDB2, whereas prolonged incubation for 6 h resulted in marked accumulation and immobilization of DDB2 at the nucleolar periphery. It should be noted that these effects were observed in cells that were not treated with UV or other exogenous DNA damaging agents. Notably, the immobilized DDB2 molecules became unavailable for initiation of GG-NER (Fig. 4c,e, and Supplementary Fig. 4a and b), thereby compromising repair of CPDs (Fig. 4b), which depend mostly on UV-DDB and proceed with slow kinetics. Although UV-DDB exhibits even higher affinity for 6-4PPs and is likely involved in repair of this type of lesion as well^22^, repair of 6-4PPs was much faster and was mostly complete before DDB2 accumulation. Even in the absence of functional DDB2, 6-4PPs can be repaired quite efficiently by GG-NER directly initiated by XPC.

In our attempts to understand the underlying mechanism, we found that depletion of the proteasome subunit PSMD14 unequivocally suppressed DDB2 accumulation. Furthermore, PSMD14 itself also accumulates at the periphery of nucleoli in the presence of proteasome inhibitors, which is highly reminiscent of the observed re-localization of DDB2. These results suggest that inhibition of proteasome activity induces sequestration of proteasome components in the nucleus, which then may nucleate accumulation of other proteins, including DDB2. Considering that the 20S core particle is the target of proteasome inhibitors and that the 19S regulatory particle is required for unfolding and deubiquitylation of protein substrates to be degraded, the 26S proteasomes choked with substrates may form aggregates at the nucleolar periphery and trap various nuclear proteins including DDB2. Because loss of PSMD14 seems to lead to destabilization of the entire 19S regulatory particle (Fig. 5a), ubiquitylated protein substrates cannot be delivered to the 20S core particles any more, which rationalizes the ineffectiveness of proteasome inhibitors.

Multiple nuclear proteins are regulated by the UPS-mediated proteolysis. Notably, treatment with proteasome inhibitors induces aggregation of proteasome targets and poly-adenylated RNAs in the nucleoli^52^. These characteristic nuclear bodies, designated as nucleolar aggregates, contain DNA damage–responsive factors, such as ATM and Ku80, in addition to cell cycle regulators, transcription factors, histone modifiers, and ubiquitin. Although the subnuclear accumulation of DDB2 and PSMD14 that we observed is reminiscent of nucleolar aggregates, they differ in several respects. First, treatment with proteasome inhibitors induced accumulation of those proteins spatially close to, but excluded from the nucleoli. Therefore, DDB2 and PSMD14 do not accumulate within the nucleoli themselves under such conditions. Second, formation of nucleolar aggregates was compromised by treatment with ActD, implying that integrity of nucleolar functions is required. By contrast, MG132-induced re-localization of DDB2 and PSMD14 was stimulated by ActD, resulting in merged localization with nucleolar markers. Because both proteins were still excluded from the nucleoli in cells treated with ActD alone, these results suggest that functions of the proteasome and nucleoli may cooperate to regulate nucleolar localization of these proteins. Although precise roles for such aggregates in the nucleus remain to be understood, this process might be implicated in formation of intranuclear inclusion bodies associated with some neurodegenerative diseases^64^. Further studies will be necessary to understand the function and fate of proteasome aggregates.

Intriguingly, we found that depletion of PSMD14 severely compromised recruitment of DDB2-mKO1 to local DNA damage (Fig. 6c), although the mobility of DDB2-mKO1 was unaffected (Supplementary Fig. 3d) in contrast to the effect of proteasome inhibition. This defective DDB2 recruitment could explain for selective impairment of CPD repair in the absence of PSMD14 (Fig. 6a,b). It is currently unknown how PSMD14 could influence recruitment of DDB2 to DNA damage. It seems unlikely that the proteasome complex itself has a direct binding affinity for damaged DNA, while our attempts to detect physical interactions between DDB2 and PSMD14 have been unsuccessful. Notably in this regard, it was previously reported that the 19S regulatory particle in yeast plays a proteolysis-independent role in GG-NER^65^. This function of the 19S regulatory particle depends on its ATPase activities as well as the N-terminal UbL domain of RAD23, although the precise underlying mechanism still remains to be elucidated. Considering that budding yeast does not express a DDB2 homolog, roles of the 19S regulatory particle in NER may not be exactly the same as in mammalian cells. However, we think it still possible, for instance, that the 19S regulatory particle is involved in modulation of chromatin structures that affect interactions between DNA lesions and damage sensor proteins. Because displacement of endogenous PSMD14 by mutPSMD14^R^ compromises CPD repair in a DDB2-dependent manner (Fig. 6e,f), not only ATPase, but also deubiquitylase activities of the 19S regulatory particle may be important for this function.

In conclusion, this study uncovered unprecedentedly diverse impacts of UPS dysfunctions on mammalian GG-NER (Fig. 7). In response to UV-induced DNA damage, RAD23 and the proteasome can be recruited to damaged sites in an XPC-independent manner, presumably to degrade proteins ubiquitylated by CRL4^DDB2^. However, effects of proteasome inhibition are not limited to blockage of proteolysis: especially, sustained inhibition of the proteasome results in aggregation of proteasome components at the periphery of nucleoli, by which DDB2 is trapped and sequestered from its canonical functions in GG-NER. Although depletion of the 19S regulatory particle alleviates such DDB2 immobilization, this in turn compromises recruitment of DDB2 to DNA damage sites. Because all of these proteasome dysfunctions impair GG-NER of CPDs, but not 6-4PPs, the structural and functional integrity of the proteasome is crucial for the UV-DDB–mediated lesion recognition sub-pathway, but not for direct initiation by XPC. Further studies are required to understand the precise roles of the proteasome in GG-NER.

## Materials and methods

### Cell culture and transformation

Human osteosarcoma U2OS cells were cultured at 37 °C in a humidified atmosphere containing 5% CO_2_ in Dulbecco’s modified Eagle medium (Nissui Pharmaceutical, Tokyo, Japan) supplemented with 10% fetal bovine serum. The bicistronic mammalian expression vector pIREShyg (Takara Bio, Kusatsu, Shiga, Japan) was used for stable expression of FLAG-XPC and DDB2-HA. The linearized construct was introduced into U2OS cells by electroporation, and stably transformed cells were selected and maintained in the culture medium containing 200 µg/ml hygromycin B (Fujifilm Wako Pure Chemical, Osaka, Japan). Transfection with the construct for EGFP-NPM expression^66^, purchased from Addgene (Watertown, MA, USA), was performed similarly, and selection was performed with 300 µg/ml G418 (Thermo Fisher Scientific, Waltham, MA, USA). The retroviral expression vector pMMP-puro was used for stable expression of mKO1-fused RAD23B, DDB2, and PSMD14. Production of recombinant retroviruses and infection of cells were performed as previously described^44^, and culture medium containing 1 µg/ml puromycin (Merck, Darmstadt, Germany) was used for selection of stable transformants.

### Treatment with siRNA

The target sequence for depletion of PSMD14 was described previously^56^, and the siRNA was synthesized by Japan Bio Services (Asaka, Saitama, Japan). The siRNA targeting DDB2 (Hs_DDB2_6, SI02780323) and control siRNA (AllStars Negative Control siRNA) were purchased from Qiagen (Hilden, Germany). For transfection of cells with siRNA, the Lipofectamine RNAiMAX reagent (Thermo Fisher Scientific) was used, and the final concentration of siRNA in the culture medium was adjusted to 20 nM. Cells were cultured for 2 days, and then subjected to further experiments.

### Gene disruption

Disruption of the endogenous *DDB2*, *XPC* and *RAD23B* gene in U2OS cells was carried out using the GeneArt CRISPR Nuclease Vector with CD4 Enrichment Kit (Thermo Fisher Scientific). The gRNAs targeted sequences within exon 5 of *DDB2* (5′-GTCTGCTAGTAGCCGAATGG-3′), exon 6 of *XPC* (5′-TAGTAGGTGTCCACATCTCGAGG-3′), and exon 8 of *RAD23B* (5′-CCTATCTGCTGTAGTAACGCTGG-3′). After enrichment with anti-CD4 magnetic beads, single clones were isolated by limiting dilution. Protein expression of DDB2 or XPC in each clone was checked by immunoblot analyses, and homozygous gene disruption was confirmed by sequencing of genomic DNA.

### Immunoblotting

To prepare cell extracts, cells were lysed for 30 min on ice with CSK buffer (10 mM PIPES–NaOH [pH 6.8], 3 mM MgCl_2_, 1 mM EGTA, 0.3 M NaCl, 10% glycerol, 0.1% Triton X-100, 50 mM NaF) or NP lysis buffer (25 mM Tris–HCl [pH 7.4], 0.3 M NaCl, 1% Nonidet P-40, 5% glycerol, 1 mM EDTA) containing protease inhibitor cocktail (0.25 mM phenylmethylsulfonyl fluoride, 1 μg/ml leupeptin, 2 μg/ml aprotinin, 1 μg/ml pepstatin, 50 μg/ml Pefabloc SC). All protease inhibitors were purchased from Merck. To detect ubiquitylation of XPC, 10 mM *N*-ethylmaleimide (Fujifilm Wako Pure Chemical) was added to the lysis buffer. Proteins were separated by SDS-PAGE and transferred onto polyvinylidene difluoride membranes (Immobilon-P, Merck). After blocking with 5% skim milk in TBS-T (50 mM Tris–HCl [pH 8.0], 150 mM NaCl, 0.1% Tween 20), the membranes were incubated with primary antibody diluted in blocking solution. Following extensive washing, the membranes were incubated with the appropriate alkaline phosphatase-conjugated secondary antibodies (Merck). Immunoreactive bands were visualized by chemiluminescence using CDP-Star (Thermo Fisher Scientific) as a substrate. Detection of chemiluminescence was carried out on a lumino-imaging analyzer (ImageQuant LAS 4010, Cytiva, Tokyo, Japan) or with X-ray films (Super RX, Fujifilm, Tokyo, Japan).

### Quantification of UV-induced photolesions in genomic DNA

Repair rates of DNA photolesions (6-4PPs or CPDs) after UVC irradiation of cells were assessed quantitatively by an enzyme-linked immunosorbent assay as described previously^44,67^. The lesion-specific antibodies 64 M-2 and TDM-2 were purchased from Cosmo Bio (Tokyo, Japan). Mean values and SEMs were calculated from at least three independent experiments, and statistical significance of the difference was assessed by Student's t-test.

### Local UVC stimulation by three-photon absorption

We used a customized confocal laser scanning microscope system (FV3000, Olympus, Tokyo, Japan), which was equipped with a 780-nm femtosecond fiber laser (CFL, Calmar Laser, Palo Alto, CA, USA). According to the principle of three-photon absorption, this system can be used to apply 260-nm UVC-like stimulation to any given area within the nucleus.

Cells expressing fluorescently-labeled proteins were seeded in 35-mm glass-bottom dishes (glass diameter 14 mm, glass thickness No. 1.5, poly-D-lysine–coated, MatTek, Ashland, NA, USA) and cultured overnight. Under the confocal laser scanning microscope, a region of interest (ROI) was set within the nucleus and stimulated with the 780-nm femtosecond laser at ~ 40% power. Fluorescence images were acquired every 10 s (mKO1-RAD23B, DDB2-mKO1) or 20 s (PSMD14-mKO1).

Data analyses were carried out using the cellSens Dimension software (Olympus). For quantification, three ROIs were defined: 1) the ROI used for stimulation (S); 2) the entire nucleus of the stimulated cell (N); and 3) an area outside the cell, used as a background (BKG). Average fluorescence intensities per pixel were measured for these ROIs (defined as F_S_, F_N_, and F_BKG_, respectively), and relative fluorescence intensities in the stimulated area (F_R_) were calculated for each time point using the following formula:

$$ {\text{F}}_{{\text{R}}} = \left( {{\text{F}}_{{\text{S}}} - {\text{F}}_{{{\text{BKG}}}} } \right)/\left( {{\text{F}}_{{\text{N}}} - {\text{F}}_{{{\text{BKG}}}} } \right) $$

The data were normalized by defining the F_R_ value prior to stimulation as 1 (except for Fig. 4d and Supplementary Fig. 3b, where absolute levels of DDB2-mKO1 accumulation are of interest). The data were collected with more than 20 cells from three independent experiments, and mean values and SDs calculated were plotted as graphs. Student's t-tests were performed to assess statistical significance of the differences at individual time points.

### Fluorescence recovery after photobleaching

Cells expressing DDB2-mKO1 were seeded in 35-mm glass-bottom dishes (glass diameter 27 mm, glass thickness No. 1S, poly-lysine–coated, Matsunami Glass, Kishiwada, Osaka, Japan) and cultured overnight. Under the confocal laser scanning microscope, a ROI was set within the nucleus, and mKO1 fluorescence was bleached for 0.175 s with 488-, 561-, and 594-nm lasers at 100% power. After bleaching, fluorescence images were acquired every 3 s with the 561-nm laser at 1% power. As a control for quantification, three images were acquired before bleaching.

For quantification, average fluorescence intensities per pixel were measured for ROIs set within the bleached area (BL), in the unbleached area of the same nucleus (UBL), and background outside the nucleus (BKG) (defined as F_BL_, F_UBL_, and F_BKG_, respectively). Relative fluorescence intensity in the bleached area (F_R_) was calculated using the following formula:

$$ {\text{F}}_{{\text{R}}} = \left( {{\text{F}}_{{{\text{BL}}}} - {\text{F}}_{{{\text{BKG}}}} } \right)/\left( {{\text{F}}_{{{\text{UBL}}}} - {\text{F}}_{{{\text{BKG}}}} } \right) $$

To normalize the results from different cells, F_R_ before bleaching (average of three images) was defined as 1, whereas F_R_ immediately after bleaching was defined as 0. Mean values and SDs were calculated and plotted as graphs. Student's t-tests were performed to assess statistical significance of the differences at individual time points.

### Local UVC irradiation through membrane filters and immunostaining

Cells were seeded in 35-mm glass-bottom dishes (MatTek) and incubated overnight. The cells were washed once with pre-warmed PBS, covered with polycarbonate isopore membrane filters (13-mm diameter, 5-µm pore size, Merck), and irradiated with UVC at 100 J/m^2^ under a germicidal lamp (GL-15, Toshiba, Tokyo, Japan). After the dishes were replenished with culture medium and the filters were removed, the cells were incubated for the indicated time. Fixation and immunofluorescence staining were performed essentially as described previously^68^. Alexa Fluor–labeled secondary antibodies were purchased from Thermo Fisher Scientific. Nucleoli were visualized using Nucleolus Bright Green reagent (Dojindo Molecular Technologies, Mashiki, Kumamoto, Japan), which binds RNA and emits fluorescence. Nuclear DNA was counter-stained with Hoechst 33342 or DAPI (Dojindo Molecular Technologies). Images were acquired on a confocal laser scanning microscope.

### Other materials and methods

Site-directed mutagenesis of PSMD14 was performed with the QuikChange Lightning Site-Directed Mutagenesis Kit (Agilent Technologies, Santa Clara, CA, USA) and primer sequences described previously^56^. MLN4924 (Sequoia Research Products, Pangbourne, UK), MG132 (Fujifilm Wako Pure Chemical), and lactacystin (Peptide Institute, Ibaraki, Osaka, Japan) were purchased from the indicated suppliers. Generation of antibodies against XPC^69^, RAD23B^30^, and PSMD4^57^ was described previously. The following antibodies were purchased from manufacturers: XPC (NB100-477, Novus Biologicals, Centennial, CO, USA), DDB2 (AF3297, R&D Systems, Minneapolis, MN, USA), CUL4A (14851-1-AP, Proteintech, Rosemont, IL, USA), multi-ubiquitin (FK2; D058-3, Medical & Biological Laboratories, Nagoya, Japan), mKO2 (PM051M, Medical & Biological Laboratories), PSMD14 (ab109123, abcam, Cambridge, UK), RAD23A (24555, Cell Signaling Technology, Danvers, MA, USA), α-tubulin (T5168, Merck), and lamin B1 (C-20, Santa Cruz Biotechnology, Santa Cruz, CA, USA).

## Supplementary information

Supplementary Information.
