# Supplementary material for: Functional impacts of the ubiquitin–proteasome system on DNA damage recognition in global genome nucleotide excision repair
Source: Sci Rep. 2020 Nov 12;10:19704. doi: 10.1038/s41598-020-76898-2 (PMC7665181; doi:10.1038/s41598-020-76898-2)
Supplement: Supplementary file 1 — Supplementary Information. [file 41598_2020_76898_MOESM1_ESM.pdf]

Supplementary information

Functional impacts of the ubiquitin–proteasome system on DNA damage recognition in global genome nucleotide excision repair

Wataru Sakai[1,2], Mayumi Yuasa-Sunagawa[1], Masayuki Kusakabe[1], Aiko Kishimoto[1,2], Takeshi Matsui[1,2], Yuki Kaneko[1,2], Jun-ichi Akagi[1,3], Nicolas Huyghe[4], Masae Ikura[5], Tsuyoshi Ikura[5], Fumio Hanaoka[6], Masayuki Yokoi[1,2] & Kaoru Sugasawa[1,2] *

[1]Biosignal Research Center, and [2]Graduate School of Science, Kobe University, Kobe 657-8501, Japan. [3]Division of Pathology, National Institute of Health Sciences, Kawasaki 210-9501, Japan. [4]Institute of Experimental and Clinical Research, Catholic University of Louvain, 1200 Woluwe-Saint-Lambert, Brussels, Belgium. [5]Radiation Biology Center, Graduate School of Biostudies, Kyoto University, Kyoto 606-8501, Japan. [6]National Institute of Genetics, Mishima, Shizuoka 411-8540, Japan.

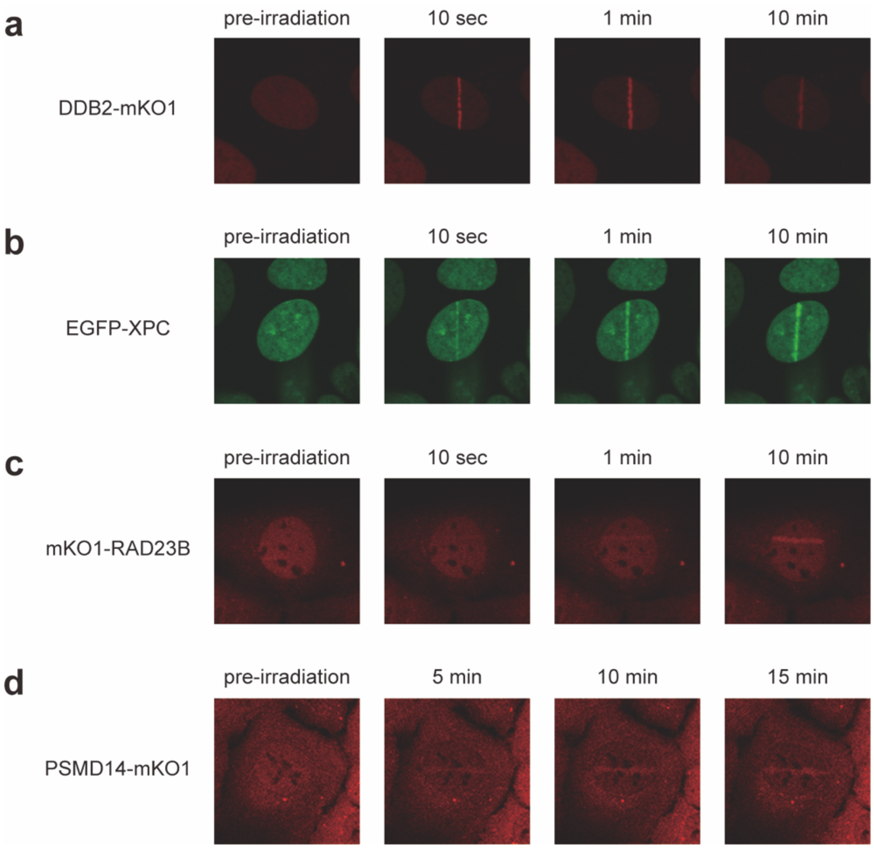

Supplementary Fig. 1 Recruitment of GG-NER and UPS factors to local DNA damage. U2OS cells stably expressing DDB2-mKO1 (**a**), EGFP-XPC (**b**), mKO1-RAD23B (**c**), or PSMD14-mKO1 (**d**) were subjected to local DNA damage induced by 780-nm femtosecond laser irradiation. Images were acquired from live cells before irradiation or at the indicated times after irradiation.

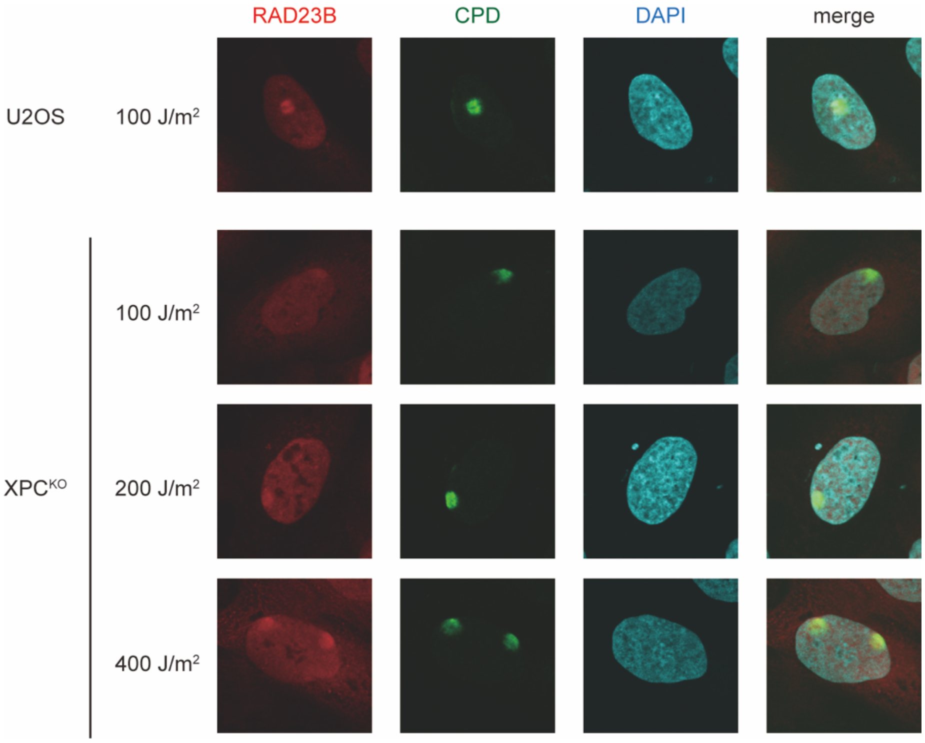

Supplementary Fig. 2 Recruitment of RAD23B to local UVC damage. U2OS cells expressing mKO1-RAD23B of the wild-type or XPC^{KO} background were irradiated with UVC at indicated doses through isopore membrane filters. After incubation for 10 min, the cells were fixed at room temperature for 15 min with the solution containing 2% paraformaldehyde and 0.25% Triton X-100, co-stained with anti-RAD23B and anti-CPD antibodies, and visualized using Alexa Fluor 594– and 488–labeled secondary antibodies, respectively. Nuclear DNA was counterstained with DAPI.

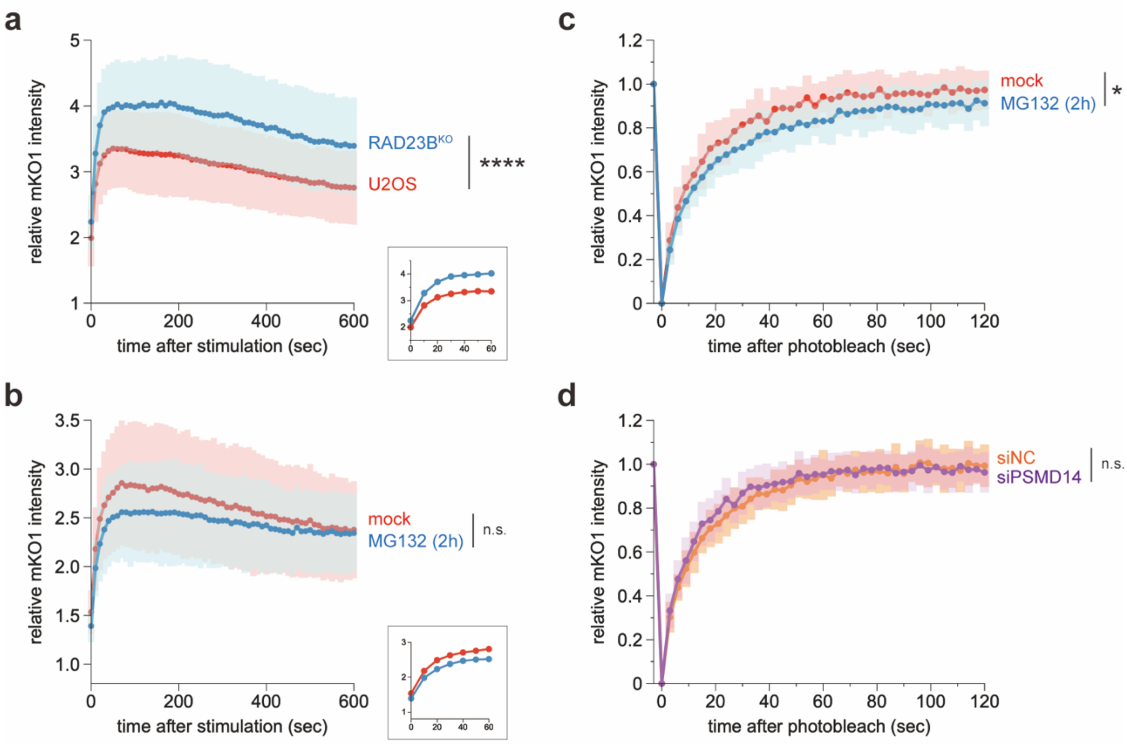

Supplementary Fig. 3 Effects of various UPS factors on intracellular dynamics of DDB2-mKO1. **a** Recruitment of DDB2-mKO1 to local DNA damage was quantitatively assessed in U2OS cells with wild-type or RAD23B^{KO} background. Inset shows enlargement of the graph in the early time range. The statistical significance assessed for the last time point is shown. **** $P < 1 \times 10^{-4}$. **b** Recruitment of DDB2-mKO1 to local DNA damage in U2OS cells, which were mock-treated or pre-treated for 2 h with 5 µM MG132. The difference was not statistically significant at any time point. **c** The DDB2-mKO1–expressing U2OS cells treated as in **b** were subjected to FRAP analyses. The total numbers of cells examined were 37 (mock) and 35 (MG132). The difference was statistically significant at all time points. * $P < 0.05$. **d** FRAP analyses of the DDB2-mKO1–expressing cells treated with siNC or siPSMD14. The total numbers of cells examined were 31 (siNC) and 29 (siPSMD14).

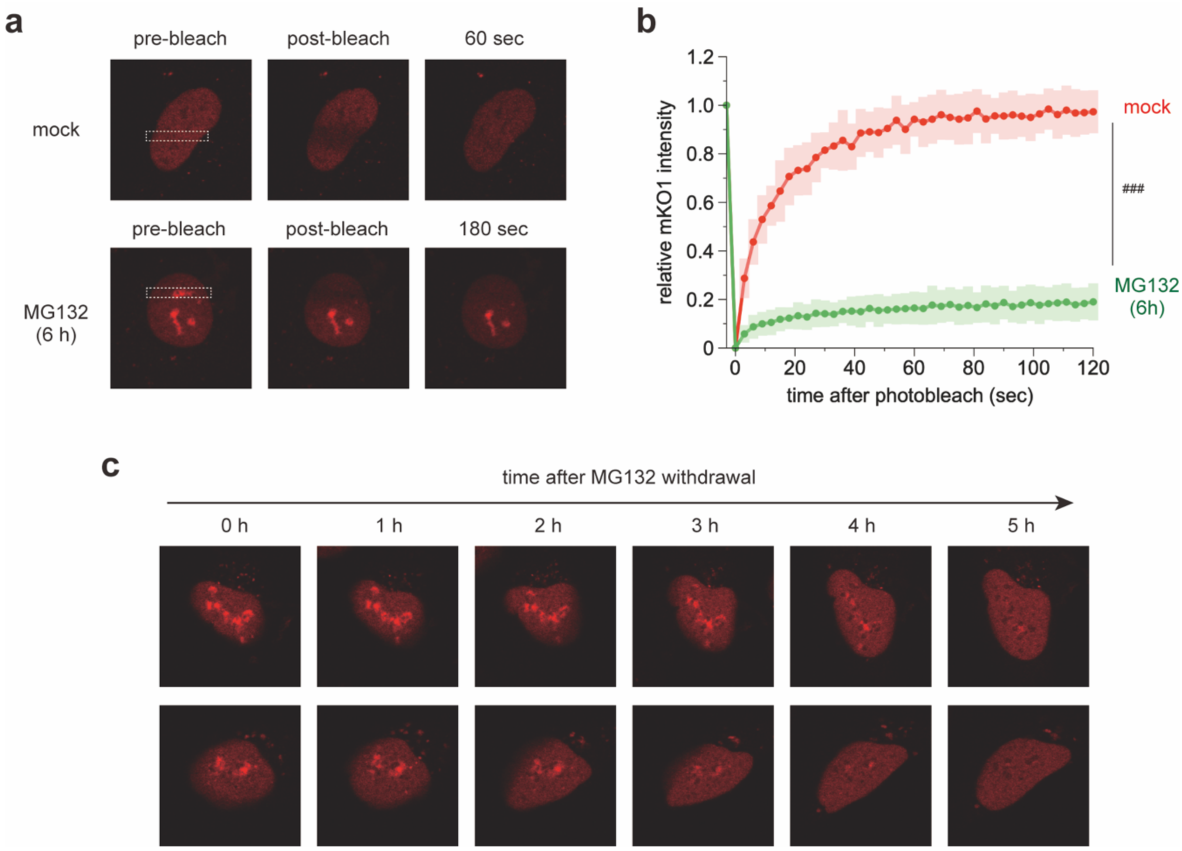

Supplementary Fig. 4 MG132 induces immobile but reversible accumulation of DDB2. a U2OS cells expressing DDB2-mKO1 were mock-treated or treated for 6 h with 5 μM MG132, and then subjected to FRAP analyses. Live-cell images acquired at the indicated times are shown. Dotted square superimposed on the pre-bleach image indicates the bleached area. **b** Quantitative representation of the FRAP analyses. For the MG132-treated cells, the areas with DDB2-mKO1 accumulation were bleached and then fluorescence intensities were monitored. Data were collected from 30 cells in total and superimposed on the data of mock-treated cells shown in Supplementary Fig. 3c. The statistical significance assessed for the last time point is shown. ### $P < 1 \times 10^{-20}$. **c** After a 6-h treatment with 5 μM MG132, the drug was removed from the culture medium and time-lapse imaging was carried out.

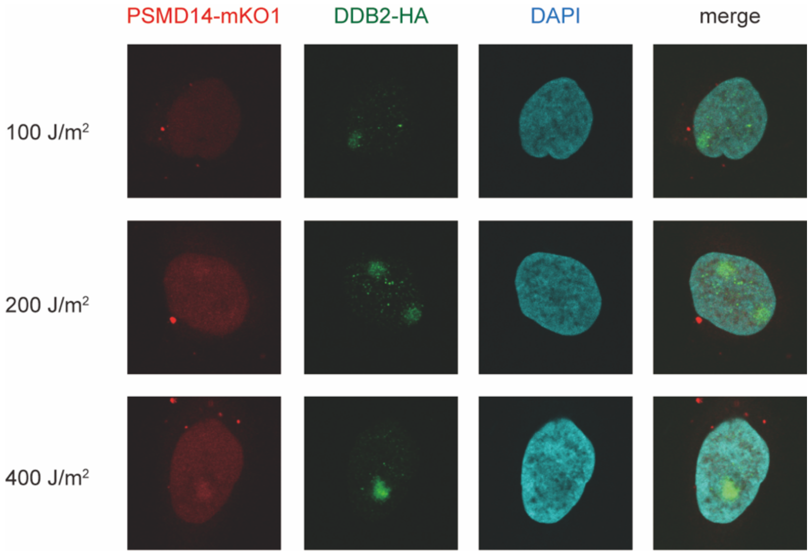

Supplementary Fig. 5 PSMD14 colocalizes with DDB2 at local DNA damage. DDB2^{KO} cells stably expressing PSMD14-mKO1 and DDB2-HA (used in Fig. 5d and e) were irradiated with UVC at indicated doses through isopore membrane filters. After incubation for 15 min, the cells were fixed as in Supplementary Fig. 2 and co-stained with anti-mKO2 and anti-HA antibodies.

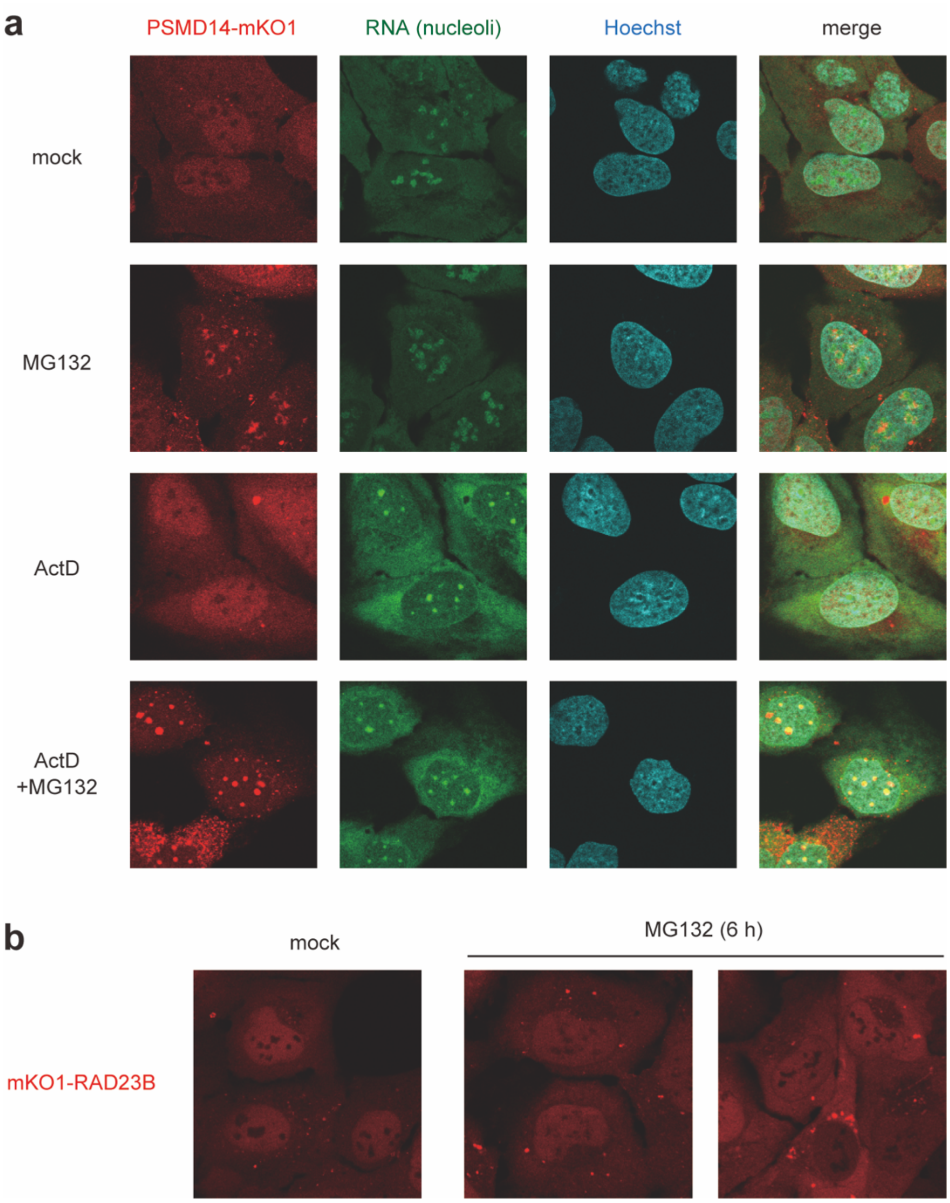

Supplementary Fig. 6 Similar patterns of MG132-induced accumulation of PSMD14 and DDB2. **a** U2OS cells expressing PSMD14-mKO1 were mock-treated or treated with 5 µM MG132 and/or 40 nM ActD as shown in Fig. 3. After fixation of the cells, the nucleoli were visualized with RNA-binding fluorescent dye (Nucleolus Bright Green). Nuclear DNA was counterstained with Hoechst 33342. **b** U2OS cells stably expressing mKO1-RAD23B were incubated for 6 h with or without 5 µM MG132 and observed under the confocal laser scanning microscope.

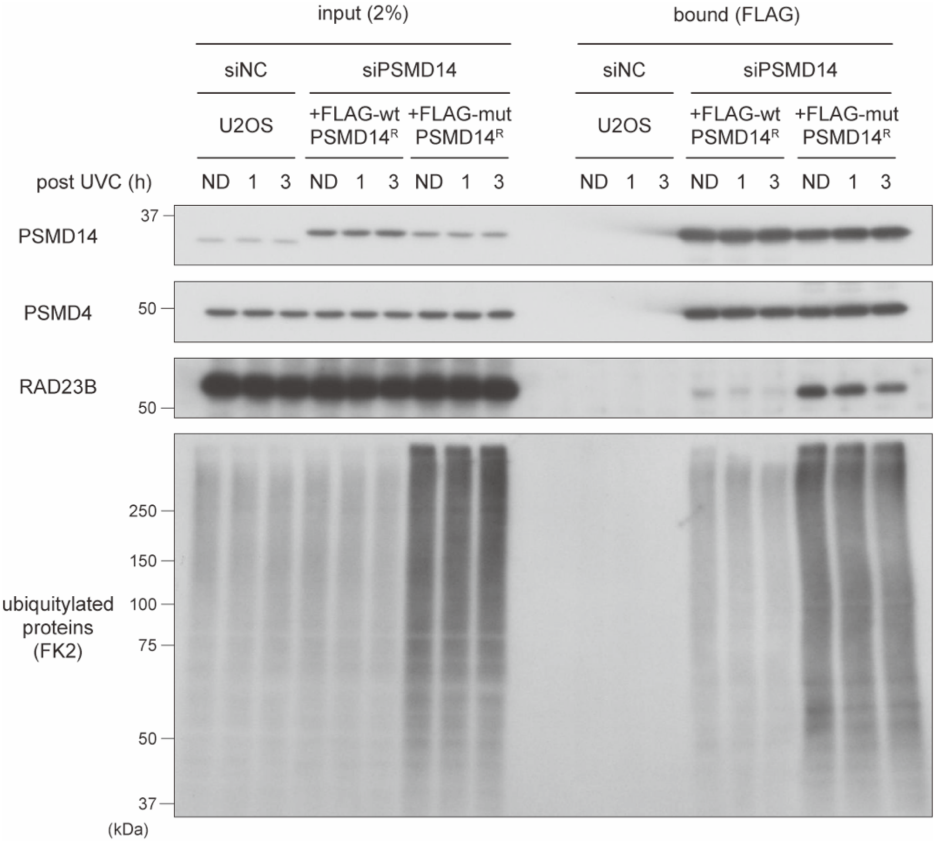

Supplementary Fig. 7 PSMD14 interacts with RAD23B and ubiquitylated proteins. U2OS cells ectopically expressing FLAG-tagged wtPSMD14^R or mutPSMD14^R were treated with siRNA to deplete endogenous PSMD14. These cells as well as control U2OS cells were irradiated with UVC at 10 J/m^2 and incubated for the indicated times (ND: unirradiated control cells). After PSMD14^R was pulled down from soluble cell extracts with the anti-FLAG antibody, co-precipitation of indicated proteins was assessed by immunoblot analyses.

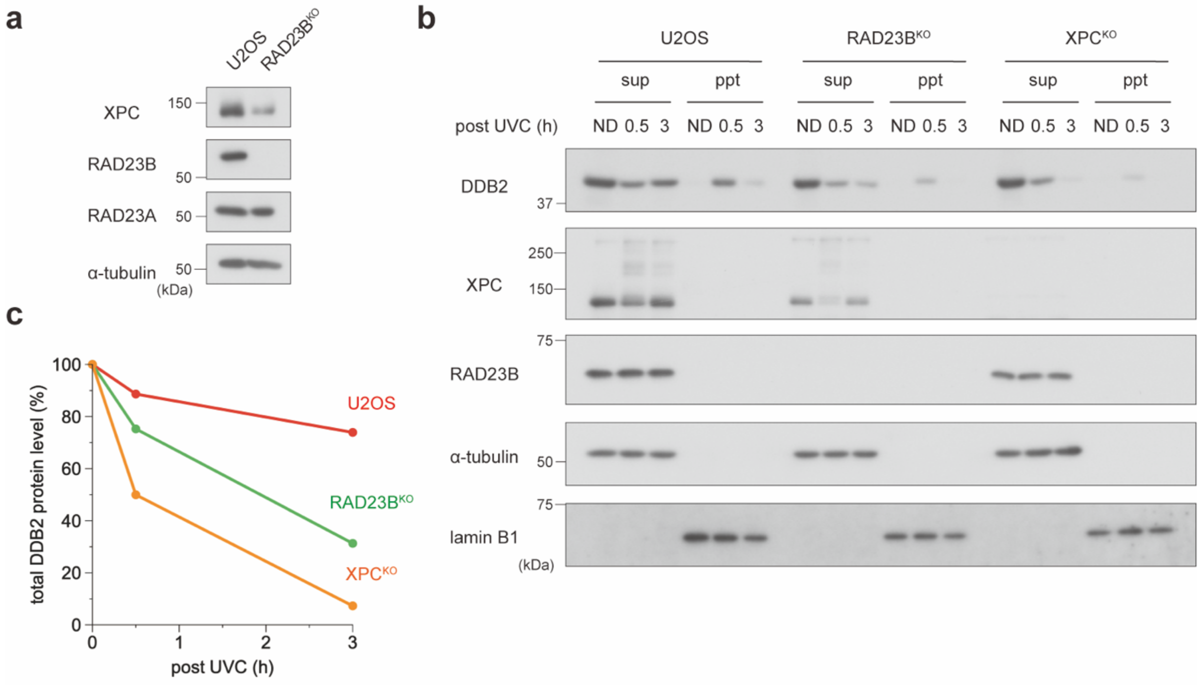

Supplementary Fig. 8 Effect of RAD23B on stability of XPC and DDB2. a Immunoblot analyses of the RAD23B^{KO} cells validating depletion of RAD23B and reduced expression of XPC. **b** U2OS, RAD23B^{KO}, and XPC^{KO} cells were irradiated with UVC at 10 J/m^2 and incubated for the indicated times (ND: unirradiated control cells). Soluble cell extracts (sup) and insoluble fractions containing chromatin-bound proteins (ppt) were subjected to immunoblot analyses using the indicated antibodies. **c** The band intensity of DDB2 was quantified for each lane of **b** with the lumino-imaging analyzer, and the total DDB2 protein levels (sup plus ppt) are plotted.

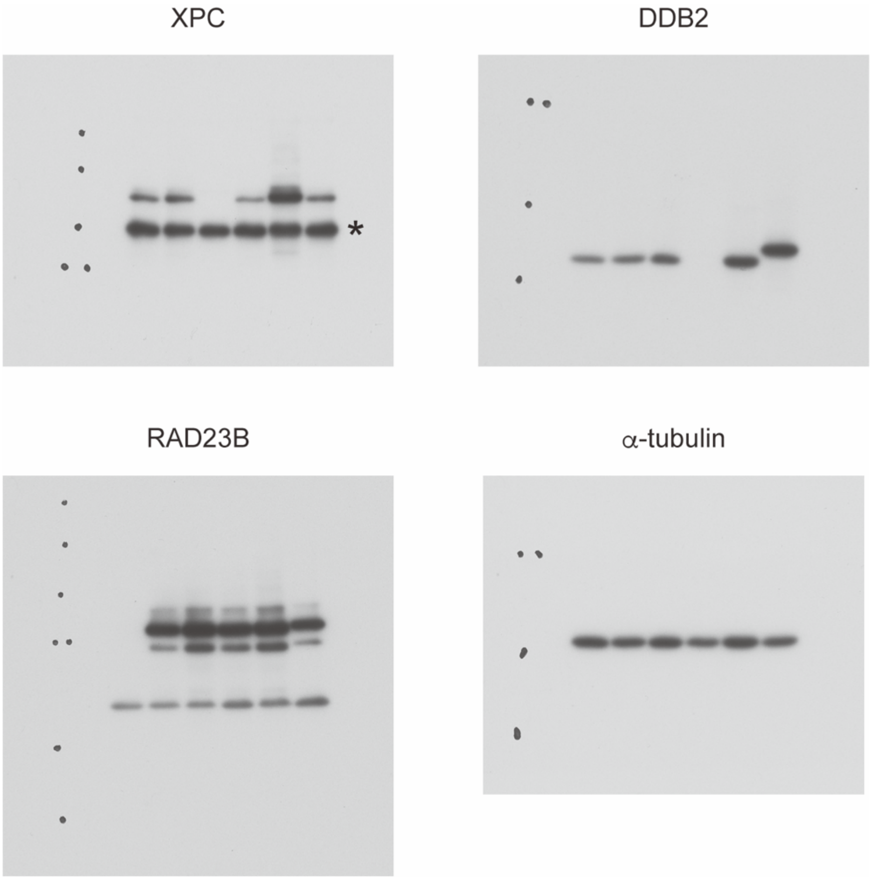

Supplementary Fig. 9 Full-length images of the blots used in Fig. 1a. All images were acquired by scanning of X-ray films. The bands indicated by asterisk are non-specific reactions of the anti-XPC antibody and omitted from the main figure.

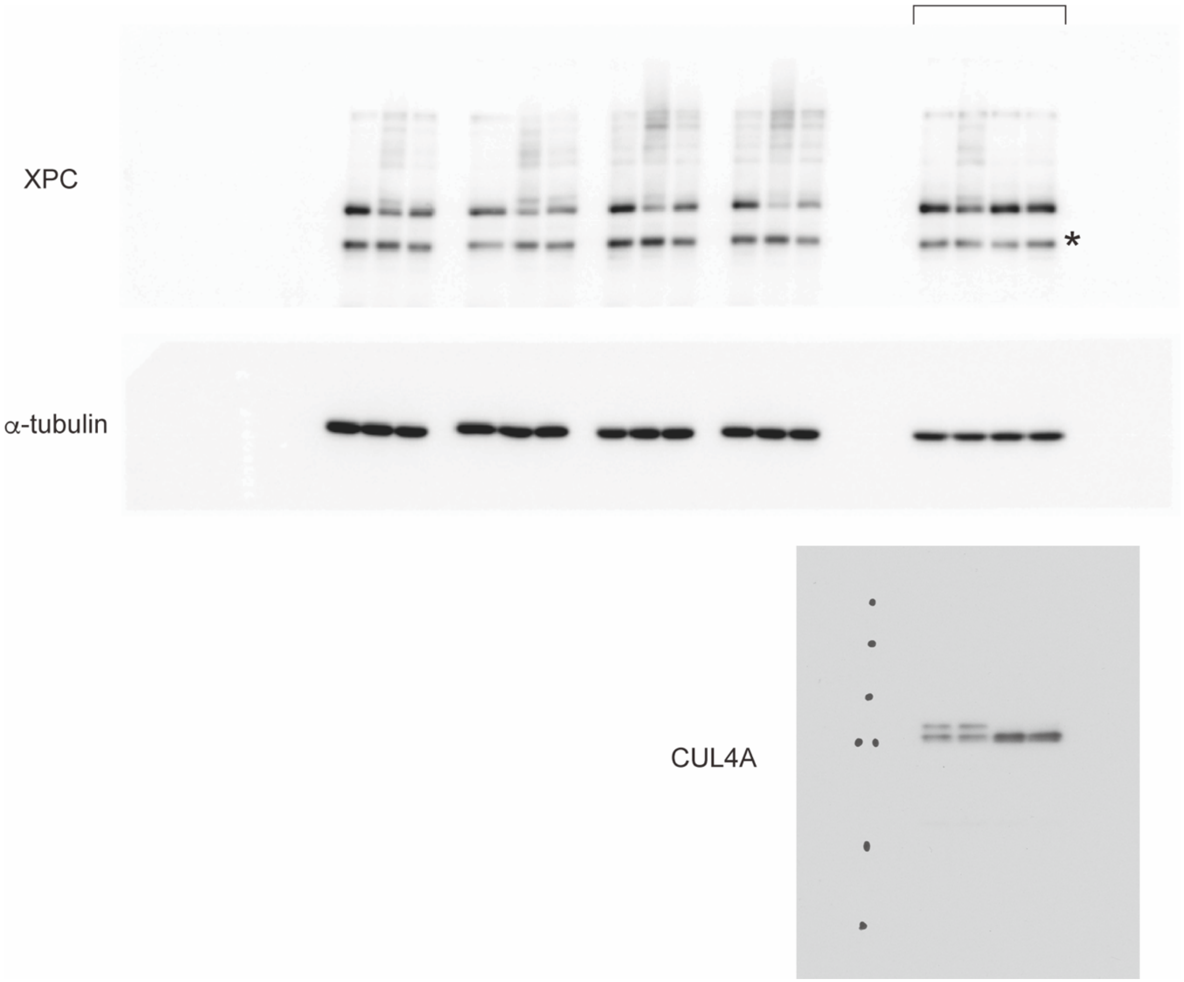

Supplementary Fig. 10 Full-length images of the blots used in Fig. 1d. For detection of XPC and α-tubulin, a blotted membrane filter was cut into two parts (higher and lower molecular weight areas), which were reacted with the individual antibodies. The four lanes on the right (indicated by a bracket) are shown in Fig. 1d (the other lanes were used for a different experiment). These images were acquired with the lumino-imaging analyzer, whereas an X-ray film was scanned for the CUL4A blot. Asterisk: non-specific reaction of the anti-XPC antibody.

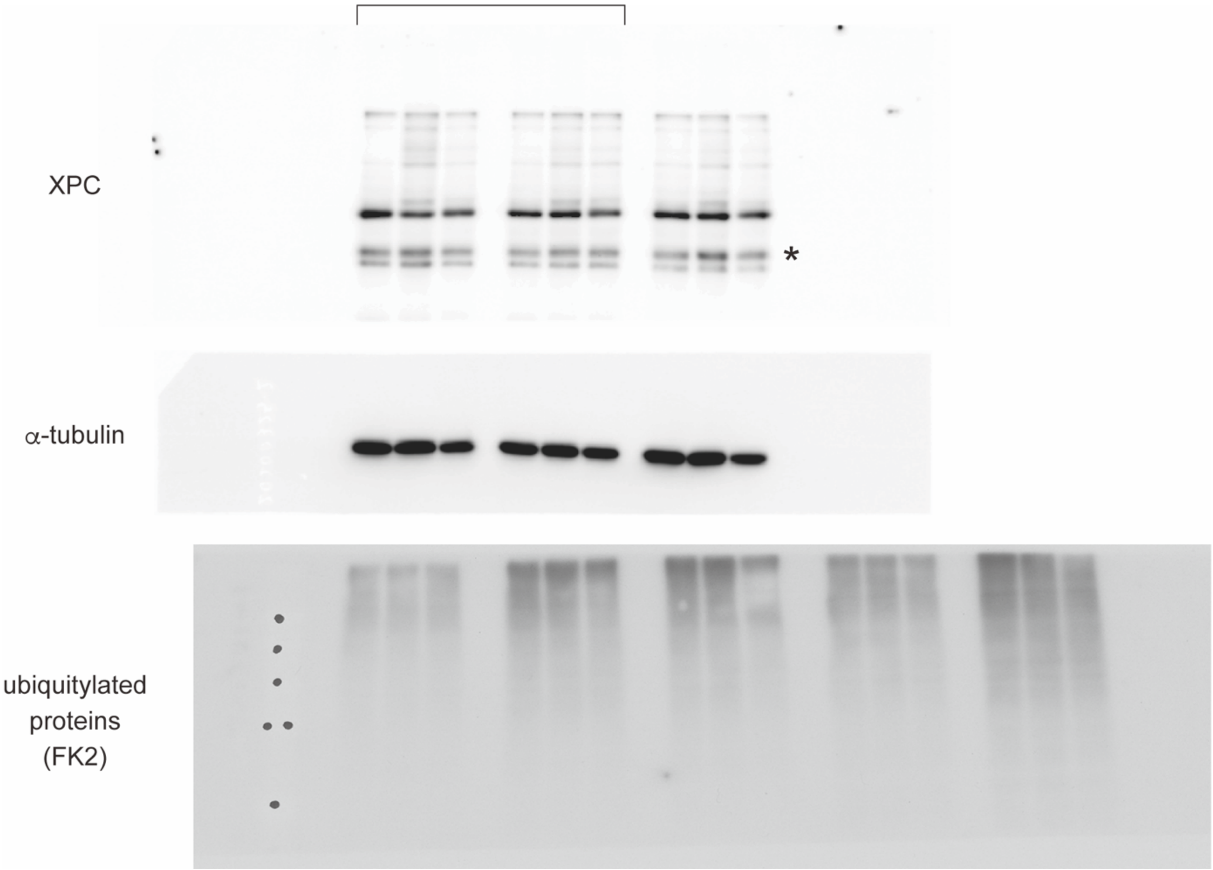

Supplementary Fig. 11 Full-length images of the blots used in Fig. 2b. The images of the XPC and α-tubulin blots were obtained as described in the legend to Supplementary Fig. 6. The six lanes on the left (indicated by a bracket) are shown in Fig. 2b (the other lanes include samples obtained with different conditions). For the ubiquitylated protein (FK2) blot, the image was acquired by scanning of an X-ray film. Asterisk: non-specific reaction of the anti-XPC antibody.

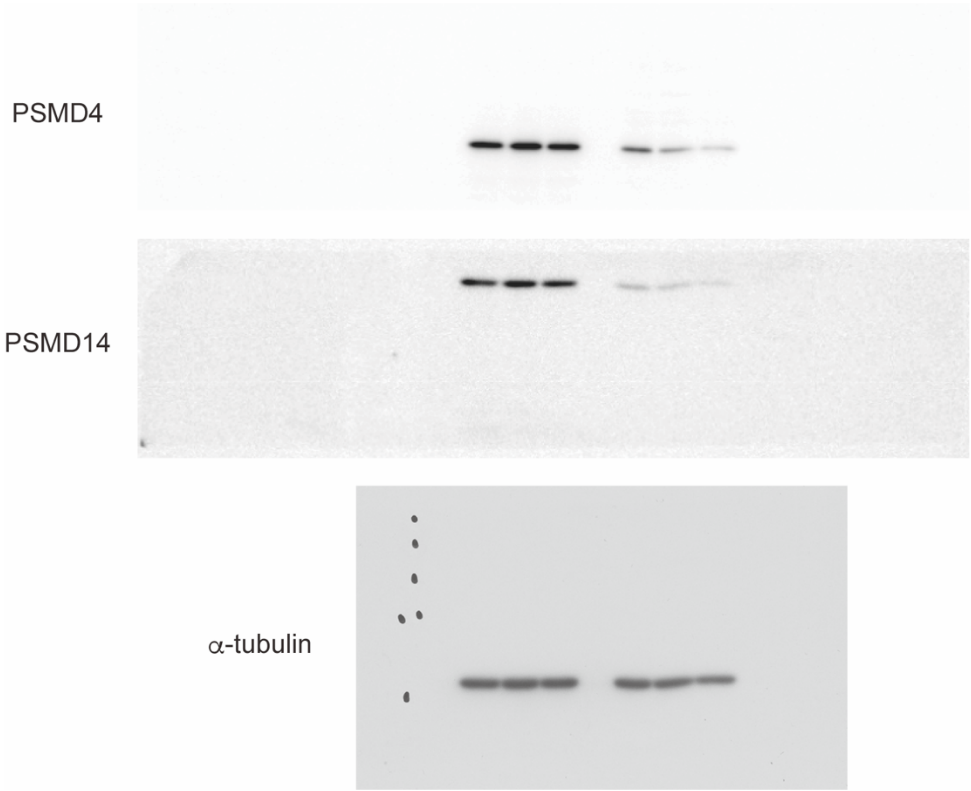

Supplementary Fig. 12 Full-length images of the blots used in Fig. 5a. For detection of PSMD4 and PSMD14, a blotted membrane filter was cut into two parts, which were reacted with the individual antibodies. These images were acquired with the lumino-imaging analyzer, whereas an X-ray film was scanned for the α-tubulin blot.

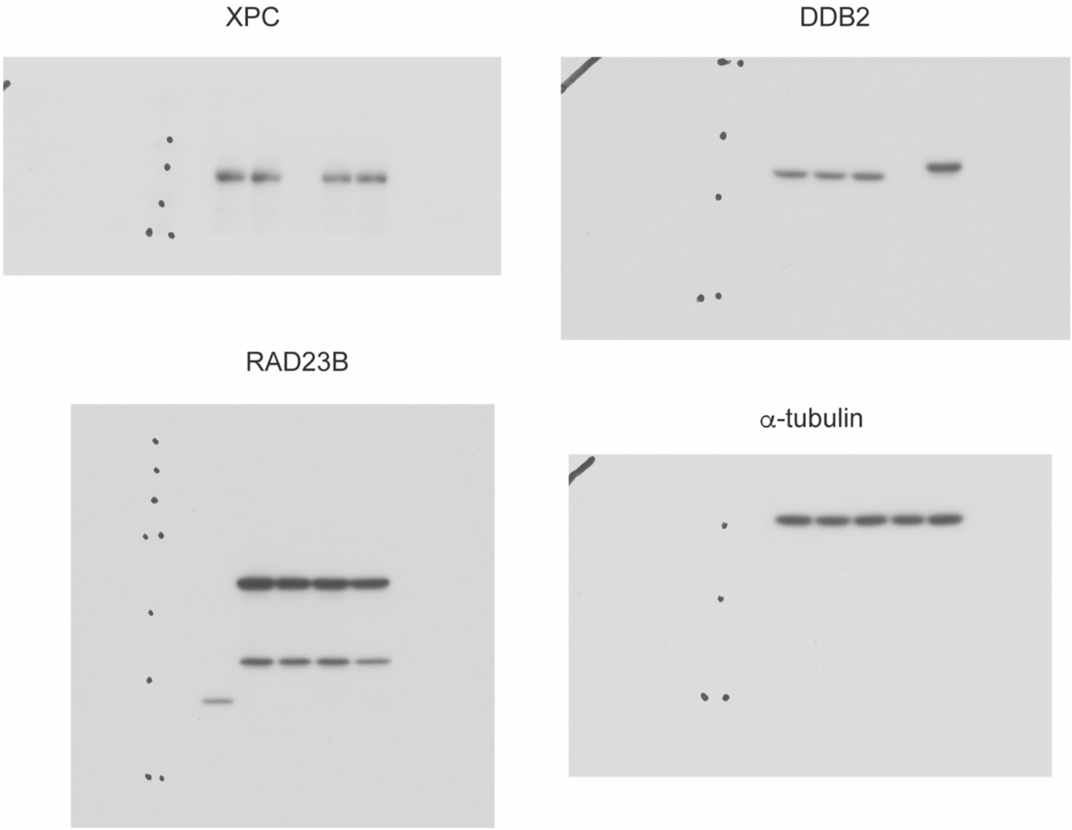

Supplementary Fig. 13 Full-length images of the blots used in Fig. 5d. All images were acquired by scanning of X-ray films. Only for this experiment, the commercial anti-XPC antibody was used, so that the non-specific bands were not detected in this XPC blot.

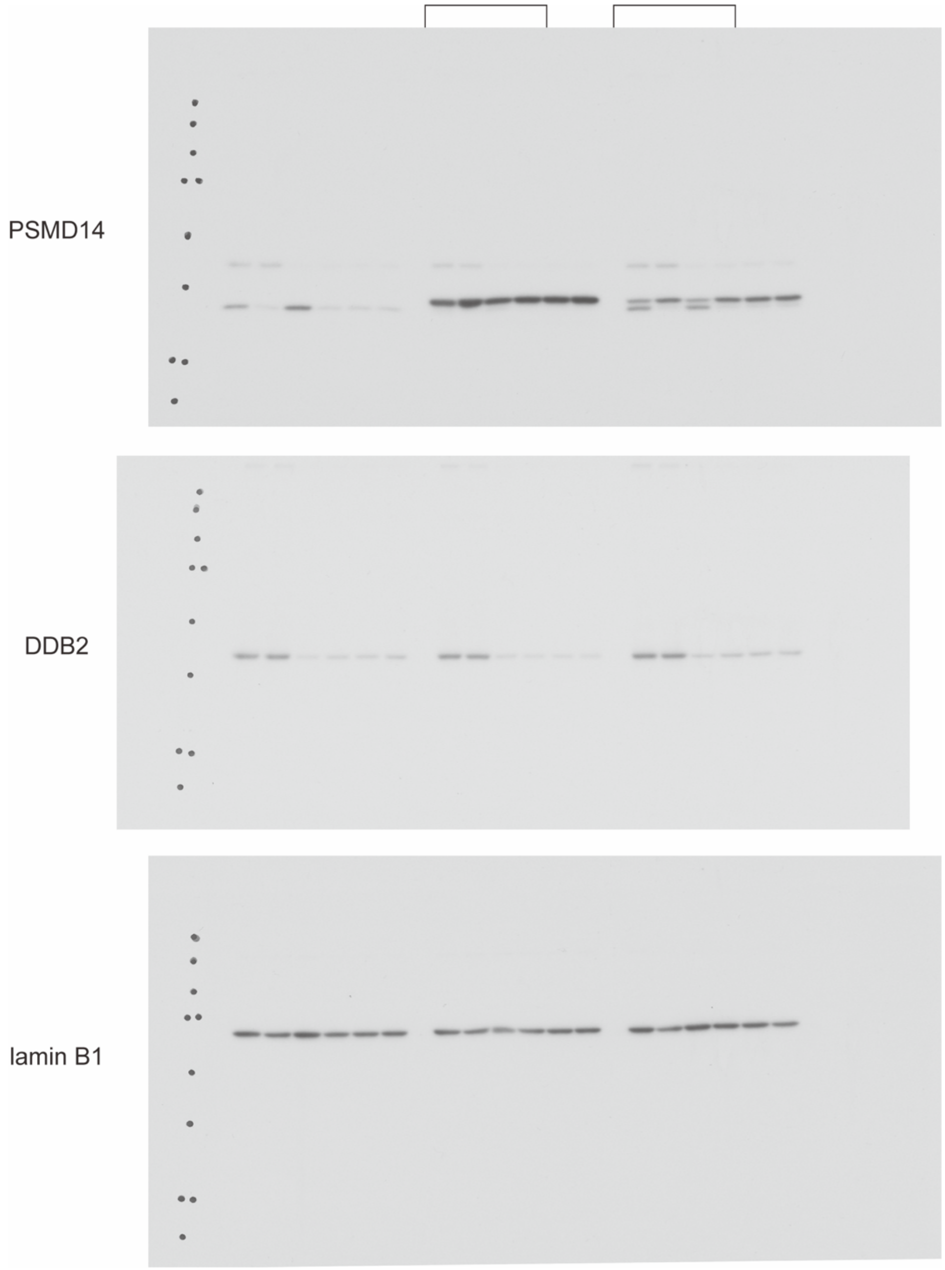

Supplementary Fig. 14 Full-length images of the blots used in Fig. 6d. All images were acquired by scanning of X-ray films. The lanes indicated by brackets are shown in Fig. 7c (the other lanes include control samples from parental U2OS cells or with different concentrations of the transfection reagent).

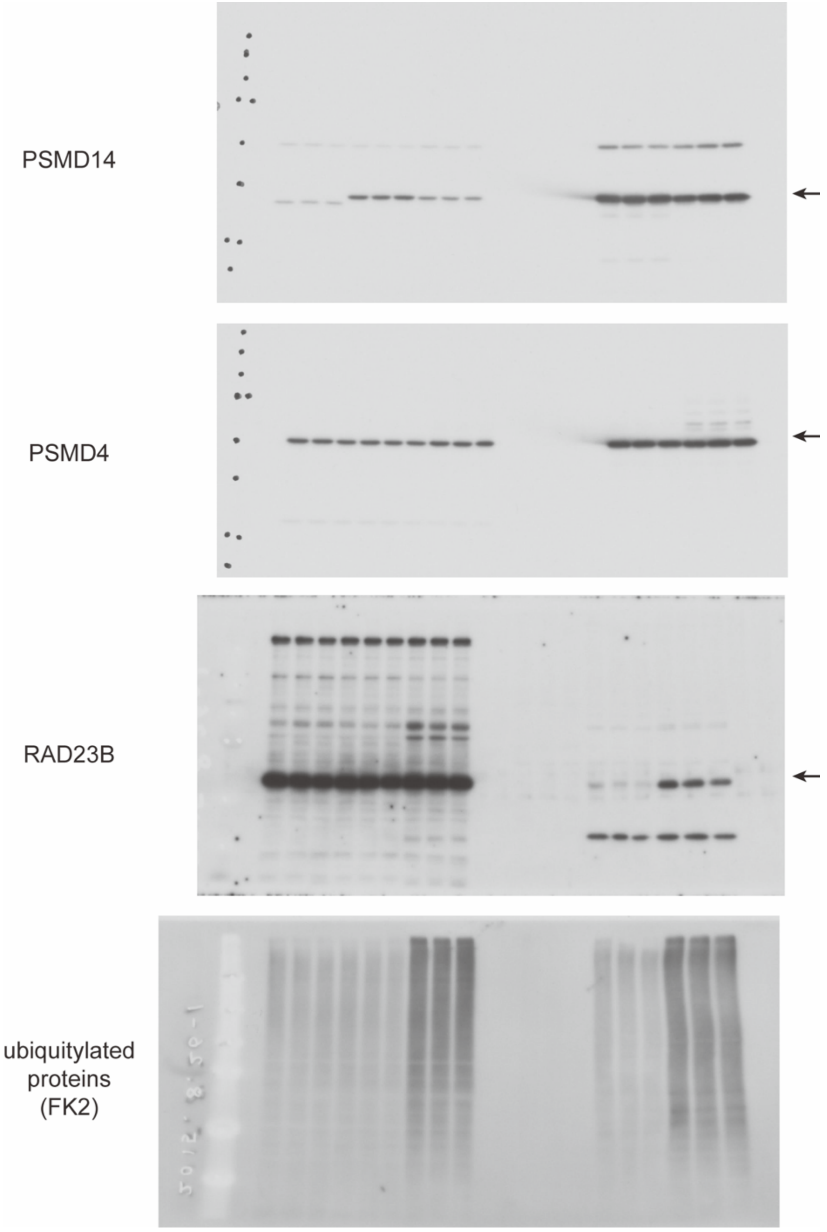

Supplementary Fig. 15 Full-length images of the blots used in Supplementary Fig. 7. All images were acquired by scanning X-ray films. Bands of the interested proteins are indicated by arrows.

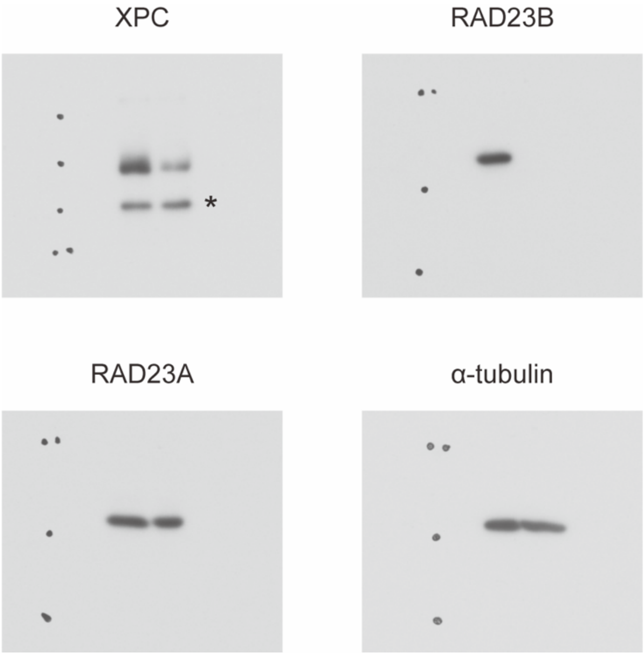

Supplementary Fig. 16 Full-length images of the blots used in Supplementary Fig. 8a. All images were acquired by scanning X-ray films. Asterisk indicates non-specific reaction of the anti-XPC antibody.

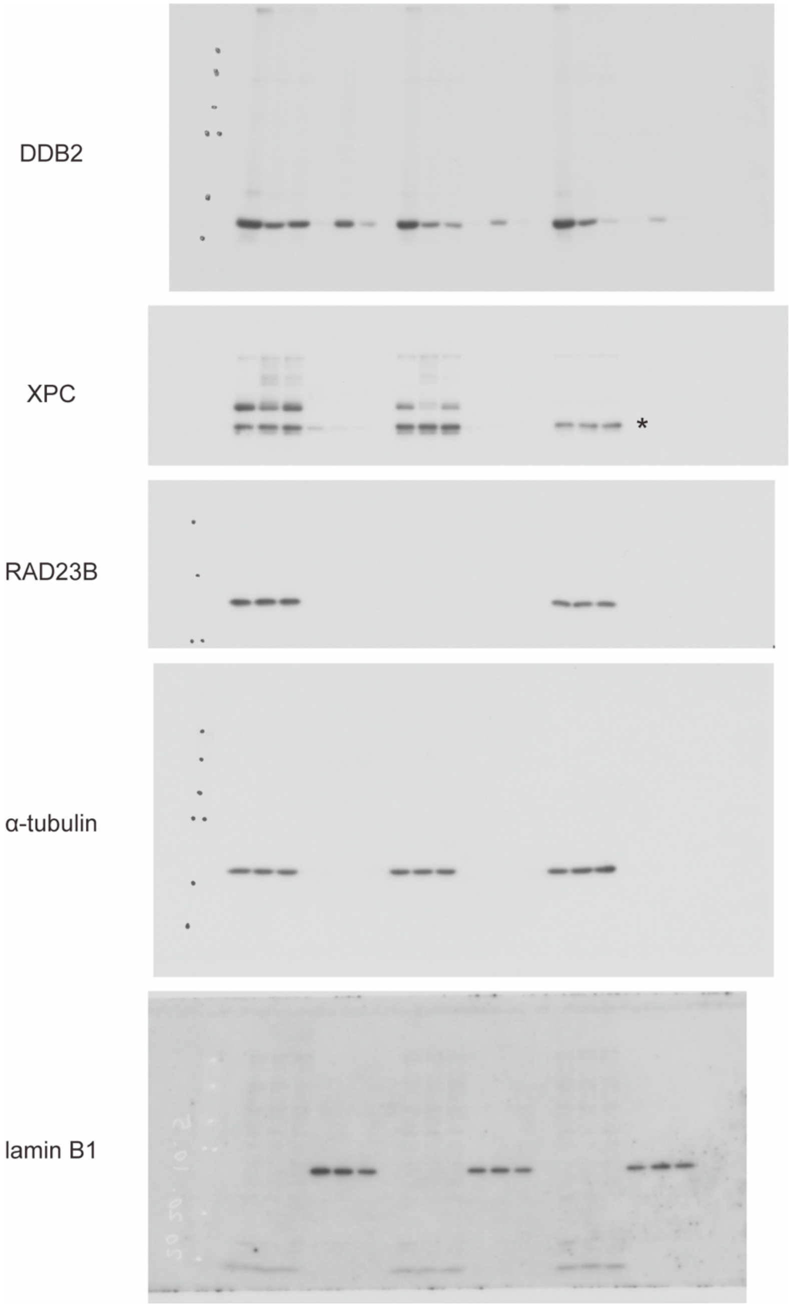

Supplementary Fig. 17 Full-length images of the blots used in Supplementary Fig. 8b. All images were acquired by scanning X-ray films. Asterisk indicates non-specific reaction of the anti-XPC antibody.
